# Drift, dispersal limitation, and homogeneous selection as key processes shaping prokaryotic community assembly in marine sediments

**DOI:** 10.1093/ismeco/ycaf189

**Published:** 2025-10-23

**Authors:** Diana Carolina Duque-Castaño, Fabiana S Paula, Brendan J M Bohannan, Alice de Moura Emilio, Julio Cezar Fornazier Moreira, Alberto G Figueiredo, Renato S Carreira, Frederico Pereira Brandini, Daniel L Moreira, Célio Roberto Jonck, Vivian Helena Pellizari

**Affiliations:** Instituto Oceanográfico, Universidade de São Paulo, Praça do Oceanográfico, 191, São Paulo, SP 05508-120, Brazil; Instituto do Mar, Universidade Federal de São Paulo, São Paulo, Brazil; Institute of Ecology and Evolution, University of Oregon, Eugene, Oregon, United States; Instituto Oceanográfico, Universidade de São Paulo, Praça do Oceanográfico, 191, São Paulo, SP 05508-120, Brazil; Instituto Oceanográfico, Universidade de São Paulo, Praça do Oceanográfico, 191, São Paulo, SP 05508-120, Brazil; Universidade Federal Fluminense, Niterói, Brazil; Pontifícia Universidade Católica do Rio de Janeiro (PUC-Rio), Rio de Janeiro, Brazil; Instituto Oceanográfico, Universidade de São Paulo, Praça do Oceanográfico, 191, São Paulo, SP 05508-120, Brazil; PETROBRAS Research Center. Centro de Pesquisas Leopoldo Américo Miguez de Mello (CENPES), Rio de Janeiro, Brazil; PETROBRAS Research Center. Centro de Pesquisas Leopoldo Américo Miguez de Mello (CENPES), Rio de Janeiro, Brazil; Instituto Oceanográfico, Universidade de São Paulo, Praça do Oceanográfico, 191, São Paulo, SP 05508-120, Brazil

**Keywords:** benthic microorganisms, marine biodiversity, marine biogeography, phylogenetic null mode, stochasticity, Southwest Atlantic

## Abstract

Marine sediment contains some of the most abundant and diverse microbial communities; however, the ecological processes shaping the benthic microbial communities at the regional scale remains poorly understood. Using a high-coverage sampling strategy, 16S rRNA gene sequencing, and ecological null models, we explored variation in the ecological processes governing benthic microbial community assembly in surface sediments across an extensive Southwest Atlantic basin. The relative importance of ecological processes varied between provinces, with drift, dispersal limitation, and homogeneous selection being the three main processes that shaped the communities. Phylogenetic bin-based analysis revealed a complex balance of assembly mechanisms, with drift dominating the majority of the bin assembly of the dominant groups such as *Candidatus* Nitrosopumilus, *Pirellula*-like planctomycetes, and *Woeseia*. The environmental factors driving this processes were associated with sediment characteristics and organic matter quality, although they differed among provinces. Drift emerged as the dominant process, influenced by sediment grain size and depth in deeper regions and organic matter properties on the continental shelf. Dispersal limitation was linked to sediment and bottom water properties, while homogeneous selection was associated with sediment aluminum and hydrocarbon content. These findings highlight the role of spatial variation and environmental factors in benthic microbial community assembly at a regional scale, providing a framework for understanding microbial community assembly in oceanic basins, and emphasizing the need for province-specific management strategies.

## Graphical Abstract

**Graphical Abstract:**
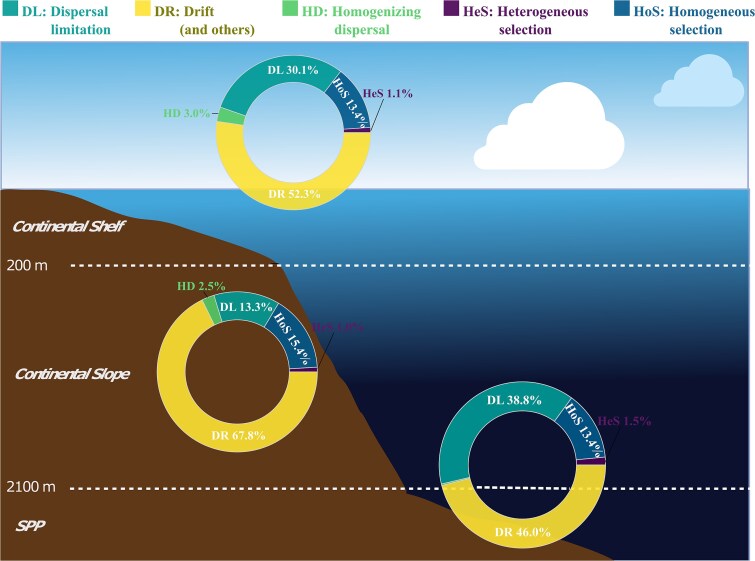

## Introduction

Marine sediment covers more than two-thirds of the surface of the Earth and harbors the largest pool of organic carbon on the planet [1]. Ecological services provided by marine sediments, such as nutrient cycling, regulation of climate-relevant gas emissions, organic matter decomposition, and carbon storage, are closely linked to the microbial communities resident in these ecosystems [2–4]. The marine sediments exhibit some of the highest compositional variation in microbial communities across the biosphere, possibly reflecting the drastic variation in marine sediment habitats and strong spatial gradients in electron donors and acceptors [5–7]. Benthic microorganisms are responsible for organic matter remineralization, nutrient cycling, and energy transfer in the sediment [8]. High-abundance groups, such as *Candidatus* Nitrosopumilus, a group of ammonia-oxidizing archaea (AOA), facilitate the first step of nitrification by converting ammonia to nitrite. Ammonia oxidation is crucial for nitrogen cycling and indirectly supports carbon sequestration by promoting primary productivity through nutrient recycling [9, 10]. Additionally, heterotrophic groups, such as *Pirellula*-like planctomycetes and *Woeseia*, contribute to organic carbon turnover by degrading complex polymers, enhancing sediment organic matter decomposition and nutrient availability [11, 12].

The nature of the sediment can be essential for the assembly of microbial communities. Properties such as pore size distribution can affect microbial activity and largely drive the assembly of the communities [13]. The sediment grain size distribution is an intuitive and integrated property that shapes the habitat of the sediment microbiome and impacts multiple biophysicochemical processes in sediments [14]. Other factors as organic matter content and quality, pore-water chemistry, sediment physicochemical, and biotic factors can influence the benthic microbial community structure and assembly [15, 16]. Understanding the assembly of marine sediment microbial communities is essential for deciphering the ecological processes that governs their composition, diversity and functioning, and for predicting how environmental changes may impact the ecological services they provide.

Vellend [17], proposed a unifying theory that synthesized the mechanisms underlying community assembly into four processes: selection, ecological drift, dispersal, and speciation. Drift refers to changes in community composition due to stochastic birth, death, and reproduction events, which can lead to divergence between communities independent of environmental factors. Dispersal limitation occurs when restricted movement of organisms between habitats limits colonization, resulting in spatially structured communities. Homogeneous selection describes deterministic processes where consistent environmental conditions across sites favor similar taxa, leading to community convergence. Together, these processes interact to determine the diversity and composition of microbial communities. The unifying framework is particularly useful because it considers the balance between determinism and stochasticity in microbial community assembly. Ning *et al.* [18] developed a framework to quantitatively infer community assembly mechanisms using phylogenetic bin-based null model analysis (iCAMP), which employs both phylogenetic and taxonomic β-diversity metrics. Recognizing that distinct assembly processes may dominate different taxa within the same community, iCAMP divided the observed taxa into various phylogenetic groupings (bins). The framework relies on the premise that phylogenetic distance could reflect niche differences within a threshold. iCAMP has been successfully applied to reveal contrasting community assembly processes for microbial communities and clades in soil, microplastics, bioaerosols, wastewater and groundwater [19–23]. However, the potential differences in assembly processes of benthic microbial communities at the regional scale, as well as environmental factors influencing their balance, remains underexplored [24].

This study represents one of the most comprehensive efforts to date to understand benthic marine microbial assembly at a regional scale. We characterized the benthic microbial community found in the surface sediment of the Santos Basin (SB), located on the southeastern Brazilian continental margin in the Southwest Atlantic Ocean. The SB is the largest offshore sedimentary basin in Brazil and contains some of the richest deepwater oil resources in the world. It is also situated near the country’s most significant urban settlements and major ports. The diverse habitats in the Atlantic Ocean, shaped by varying depths and differences in nutrient composition, make the SB an ideal location for exploring how ecological processes influence the structure of benthic microbial communities at a regional scale. We employed a spatially comprehensive grid sampling strategy, along with 16S rRNA gene amplicon sequencing using universal primers. Additionally, we utilized null models and multivariate analysis to test the following hypotheses: (i) Environmental variations among the physiographic provinces of the SB lead to spatial differences in the structure and assembly mechanisms of benthic microbial communities; (ii) The assembly of different benthic microbial groups, each with unique traits, could affect the contribution of various ecological processes in shaping their communities; (iii) Key environmental factors influencing the balance between ecological assembly processes would vary across different physiographic provinces.

## Material and methods

### Experimental site and sample

**Figure 1:**
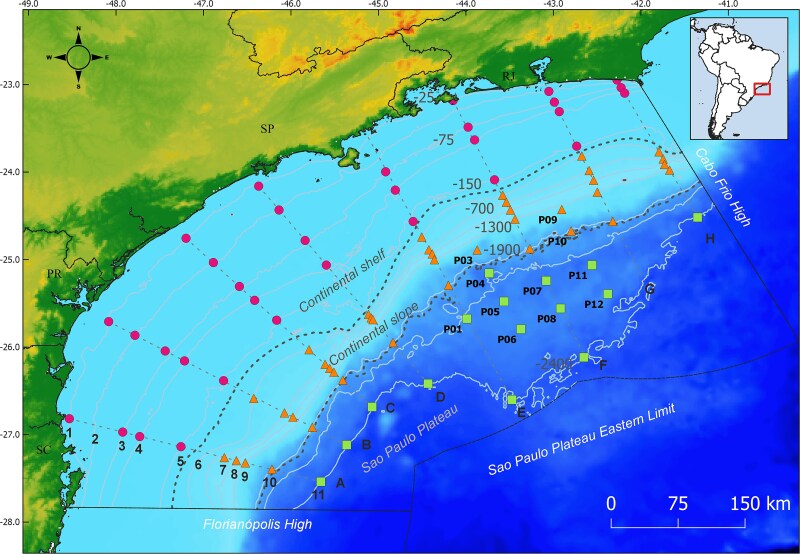
Spatial distribution of sediment sampling stations across the study area. A total of 86 stations were sampled: 75 along isobaths and 11 within presalt oil and gas fields (labeled as “P”). Stations are grouped by physiographic provinces: continental shelf (circles), continental slope (triangles), and São Paulo Plateau (SPP; squares), with provincial boundaries indicated by traced lines.

The sediment samples were collected as part of the oceanographic expeditions of the Santos Basin Regional Environmental Characterization Project (PCR-BS) [25]. The sampling grid comprised a total of 86 sediment stations, including 75 stations from eight transects following isobaths and 11 additional stations around the presalt oil and gas fields. The surface sediment samples (0–2 cm depth) were collected over the three physiographic provinces of the SB: continental shelf (32 samples), continental slope (39 samples) and the deep province of the São Paulo Plateau (SPP) (15 samples). A total of six cruises were divided into two sections: deep sea, which covered the continental slope and the SPP from June to August 2019 and the continental shelf from October to November 2019 (Fig. 1). Samples were collected using a GOMEX-type box corer with 50 × 50 cm area (0.25 m^2^), in silty and muddy sediments or a massive modified van Veen grab with 231 l (80 x 92 x 40 cm, 0.75 m^2^ surface area) in sandy sediments. For molecular analysis, samples were sterile collected from the box corer or the van Veen Grab, placed in sterile bags and stored at −80°C. Additional sediment samples were collected for chemical and geological characterization. Physical and biogeochemical data of the near the bottom water were collected using a Sea-Bird Electronics CTD [26, 27].

### DNA extraction, Illumina sequencing, and sequence processing

The genomic material from the sediment samples was extracted using the DNeasy PowerSoil Pro Kit (QIAGEN, Hilden, Germany), following the protocol provided by the manufacturer. The integrity of the DNA was evaluated using electrophoresis in a 1% TRIS-EDTA agarose gel labeled with SYBR ® Safe DNA gel stain (Invitrogen, Carlsbad, CA, USA). The DNA was quantified using a Qubit 4.0 Fluorometer with the Qubit dsDNA HS assay kit (Thermo Fisher Scientific, Waltham, WA, USA). The DNA samples were prepared for targeted sequencing with the Quick-16S NGS Library Prep Kit (Zymo Research, Irvine, CA, USA). The V4-V5 region of 16S rRNA gene was amplified using the universal primers 515FB (5′-GTGYCAGCMGCCGCGGTAA-3′) and 926R (5′-CCGYCAATTYMTTTRAGTTT-3′) [28]. The final PCR products were quantified with qPCR fluorescence readings and pooled together based on equal molarity. The final pooled library was cleaned up with the Select-a-Size DNA Clean & Concentrator (Zymo Research, Irvine, CA, USA), then quantified with TapeStation® (Agilent Technologies, Santa Clara, CA, USA) and Qubit (Thermo Fisher Scientific, Waltham, WA, USA). The final library was sequenced on Illumina MiSeq (2 × 250 pb run) (Illumina, San Diego, CA, USA).

The samples were demultiplexed at the sequencing facility according to their respective barcoded indices in the forward and reverse reads. Subsequently, sequences were processed using QIIME2 v. 2020.8 as a wrapper for the DADA2 denoising algorithm [29, 30]. Briefly, primer sequences were removed, reads quality was checked and low-quality ends were trimmed. Subsequently, the QIIME2 plugin q2-dada2 was used to denoise the sequences with the DADA2 denoising algorithm, including merging of sequences, inference of amplicon sequence variants (ASVs) and removal of chimeric reads. Finally, the sequences were classified against the SILVA138 database [31].

### Community structure analysis

We used a combination of α and β-diversity to describe the benthic community structure. After sequence processing (detailed in **Supplementary Information**), to make a comparable analysis of diversity, the overall community as well as all benthic subcommunities were subsampled to the lowest number of the total sequences. Then, the α-diversity was explored through different metrics to explore species richness and diversity. As the data did not follow a normal distribution, the Kruskal–Wallis test followed by the Dunn test were conducted to determine whether the α-diversity indices of microbial community differed significantly between physiographic provinces, isobaths, transects and near bottom water mass. The β-diversity analyses were used to identify the degree of community differentiation or the extent of change in community composition between samples. Non-metric multidimensional scaling (NMDS) based on the Bray–Curtis dissimilarity were applied to visualize the compositional variation of microbial communities across provinces. All statistical analyses were conducted in R environment v4.3.3. [32]. The significant difference in community composition between the samples was tested by analysis of similarity (ANOSIM) by the Anosim function in the Vegan 2.6.4 [33]. Tidyverse v2.0.0 was used for data processing [34] Visualizations were made using ggplot2 v3.5.1 [35]. Diversity analyses were performed using Vegan 2.6.4 and Picante v1.8.2 packages [36].

### Quantification of ecological process using null models

To quantify the relative importance of assembly processes, we applied the framework to quantitatively infer community assembly mechanisms using phylogenetic bin-based null model analysis (iCAMP) [18]. To evaluate the difference between SB physiographic provinces on ecological processes, the standardized effect size (Cohen’s d) was calculated as the difference of means between provinces divided by the combined standard deviation. The significance of difference in the relative importance of ecological processes between provinces was assessed using a permutational t-test (1000 times) and was obtained following the R code provided with the iCAMP framework. The relative contribution of each ecological process was estimated by the null models and supported by the results from the neutral community model (NCM) [37]. The NCM estimates the relationship between the site occupancy frequency of ASVs within a metacommunity and their relative abundances across the community. According to the model, abundant taxa are predicted to disperse randomly and broadly, resulting in higher occupancy frequencies, while rare taxa are more likely to be lost due to stochastic drift. The immigration rate (m) in the model reflects the extent of species distribution within the metacommunity, with higher 𝑚 values indicating greater dispersal. The overall fit of the community data to the NCM is quantified by the *R*^2^ value, which represents the proportion of community assembly driven by neutral processes. Correlation tests were conducted between groups of variables: geological, metals, hydrochemistry, hydrocarbons, fatty alcohols, fatty acids, and biological macromolecules, later the noncorrelated variables of each group in addition to hydrochemical, chemical and spatial variables were used in the microbial community analysis (see **Supplementary information** for details). Furthermore, the Mantel test was used to establish the links between the noncorrelated environmental variables and environmental factors to gain detailed insights into community assembly mechanisms.

## Results

### Spatial variability of environmental conditions

Numerous variables influence the SB surface sediment (0–2 cm) morphology, grain size, grain sorting, and carbonate content and consequently conditioning oxygen irrigation of sediment and nutrient preservation [27]. The physiographic provinces of the SB exhibited distinct sedimentological and biochemical characteristics, with variations in grain size, organic matter quantity and composition, and physicochemical conditions. The continental shelf was characterized by higher gravel content, moderate total organic carbon (TOC) concentrations, and elevated phytopigment levels in mid-shelf sediments, coupled with neutral sediment pH and low redox potential, as well as the highest carbonate content in the outer shelf. The slope displayed a high mud content, increasing carbonate content with depth, and the highest TOC concentrations. This province was marked by the most elevated redox potential, sediment pH, and hydrocarbon concentration. The SPP showed lower TOC concentrations, comparable to the shallow shelf, and increasing carbonate content with depth, as well as positive redox potential (see **Supplementary information** and Supplementary Figs S1–S8 for details).

### Microbial community composition, diversity, and variation

**Figure 2:**
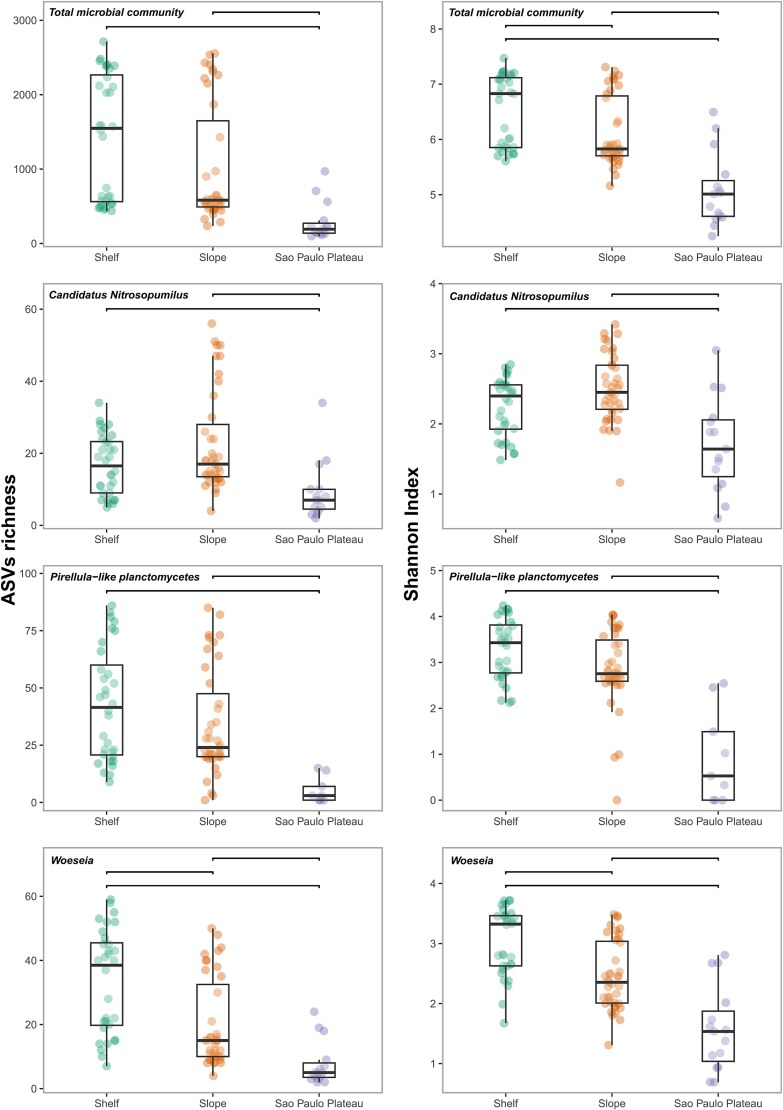
Alpha diversity of the microbial community of the SB physiographic regions for the total microbial community and *Candidatus* Nitrosopumilus, *Pirellula*-like planctomycetes (Pir4 lineage), and *Woeseia* (ASVS richness in the left column and Shannon index in the right column, the horizontal lines indicated significantly differences).

After quality control and sequence filtering the microbial community as well as subcommunities containing each of the dominant benthic microbial groups *Candidatus* Nitrosopumilus, *Pirellula*-like planctomycetes (Pir4 lineage), and *Woeseia* were defined (Supplementary Table S1)*.* To standardize sequencing depth across samples and reduce biases introduced by uneven library sizes, we applied rarefaction prior to diversity and community assembly analyses. While rarefaction may reduce the representation of low-abundance taxa and potentially underestimate the influence of stochastic processes such as drift, we opted for this approach to ensure an equal sampling effort across the samples. The rarefaction curves were drawn by showing the number of ASVs with increasing numbers of subsampled sequences. They were all saturated or nearly saturated for samples at the basin scale, as well as in every physiographic province, showing a good coverage of species richness in our study (Supplementary Fig. S9). Dominant microbial taxa were recurrently identified throughout the surface sediment of the SB. However, their abundances, including those of the major benthic groups showed variation at the different physiographic provinces (see **Supplementary Information,**  Supplementary Table S2 and Supplementary Fig. S10 and S11 for more details). The total microbial community as well as the major benthic microbial groups displayed significantly lower ASVs richness in the SPP, the spatially more restricted province of the SB, when compared to the continental shelf and slope (Kruskall–Wallis, *P* < .05). The Shannon diversity index showed that the total microbial community and *Woeseia* displayed a significantly higher α-diversity at the continental shelf when compared to the slope and SPP (Kruskall–Wallis, chi-squared = 28.29, *P* = 7.20 e-7 and chi-squared = 33.14, *P* = 6.37 e-8, respectively) (see Fig. 2). *Candidatus* Nitrosopumilus displayed the highest median of the Shannon diversity index at the continental slope, whereas *Pirellula*-like planctomycetes showed the highest median at the continental shelf, with both groups showing significantly higher Shannon indexes at the continental shelf and slope when compared to the SPP (Kruskall–Wallis, chi-squared = 17.22, *P* = 1.80 e-4 and chi-squared = 23.78, *P* = 6.85 e-6, respectively) (see **Supplementary information** and Supplementary Figs S12–S14 for details).

**Figure 3:**
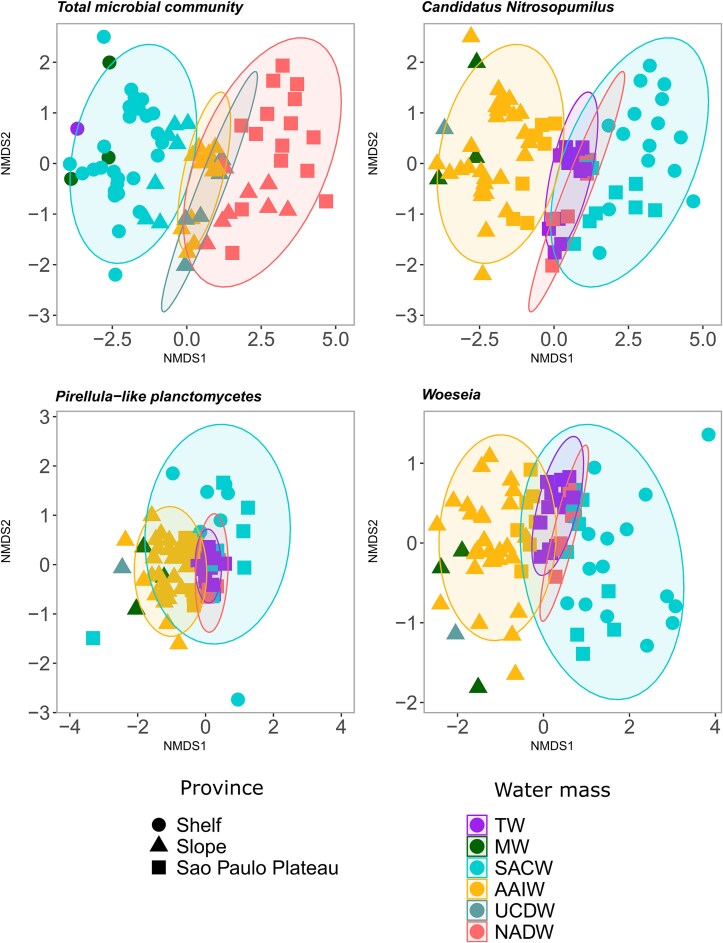
NMDS plots based on Bray–Curtis dissimilarity of surface sediment microbial communities from the Santos Basin. Ordinations were performed for the total prokaryotic community and three dominant lineages: *Candidatus* Nitrosopumilus, *Pirellula*-like Planctomycetes (Pir4 lineage), and *Woeseia*. Shapes represent physiographic provinces, and colors indicate bottom water masses: WT (tropical water), MW (mixture water), SACW (South Atlantic central water), AAIW (Antarctic intermediate water), UCDW (upper circumpolar deep water), and NADW (North Atlantic deep water). Shaded polygons represent 95% confidence ellipses for each water mass.

The microbial community structure using 16S rRNA (β-diversity) revealed that composition of the total microbial community and of the major benthic groups, Candidatus Nitrosopumilus, Pirellula-like Planctomycetes (Pir4 lineage), and Woeseia, varies with physiographic province, bottom water mass and depth as evidenced by NMDS plots based on Bray–Curtis dissimilarity (Fig. 3 and Supplementary Fig. S15) and the ANOSIM (Table 1). The NMDS plots showed clear clustering of samples according to bottom water mass and physiographic province. ANOSIM results indicated that the highest differences displayed by the total microbial community and *Pirellula*-like planctomycetes was between the physiographic provinces of the basin. By contrast, for the *Candidatus* Nitrosopumilus and *Woeseia* the highest difference were displayed between different depths (isobaths). The differences in the communities structure indicated differences in the relative importance of ecological processes for the microbial benthic community in the different physiographic provinces and isobaths of the SB.

**Table 1:** ANOSIM testing the significant difference between the microbial communities of the surface sediment of the SB by physiographic province, bottom water mass and depth. A higher ANOSIM R value indicates that groups are more different from each other.

	**Between provinces**		**Between bottom water mass**		**Between depths**	
	**ANOSIM-R**	***P***	**ANOSIM-R**	***P***	**ANOSIM-R**	***P***
Total microbial community	0.8119	.001	0.6243	.001	0.7656	.001
*Candidatus* Nitrosopumilus	0.7386	.001	0.7111	.001	0.7542	.001
*Pirellula*-like planctomycetes	0.6057	.001	0.3549	.001	0.5763	.001
*Woeseia*	0.6414	.001	0.5097	.001	0.6872	.001

### Community assembly in the different physiographic provinces

**Figure 4:**
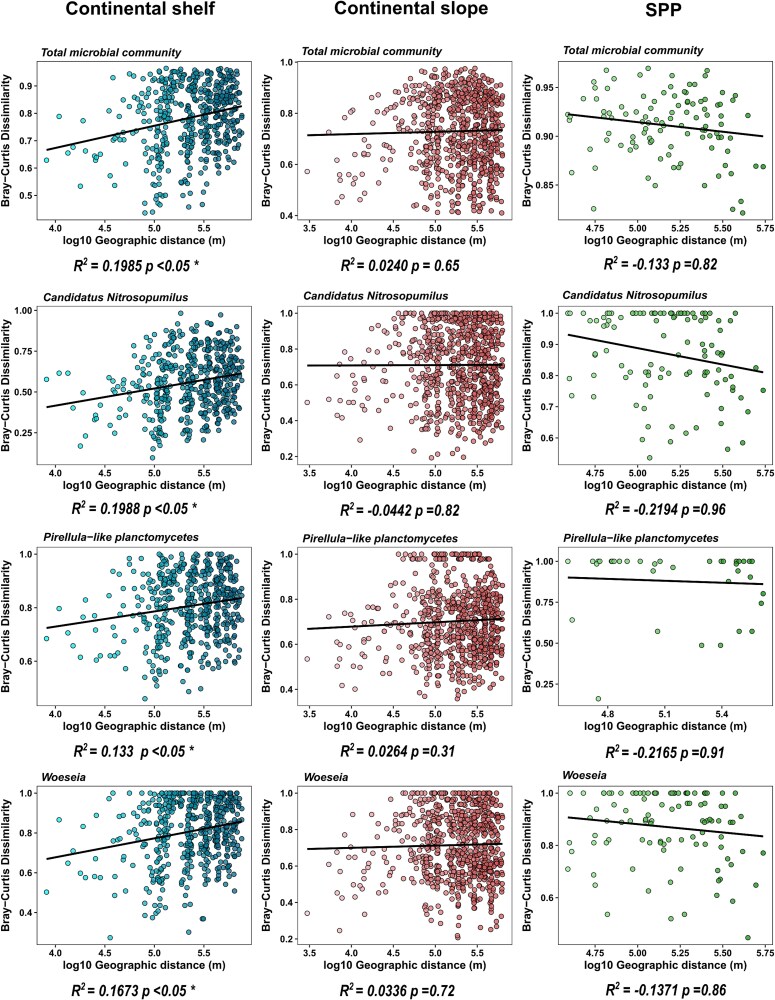
Increase in the Bray–Curtis dissimilarity of SB microbial communities with geographic distance across physiographic provinces. Significant increase in dissimilarity patterns are indicated by a ^*^. The black lines indicates the linear model fit.

The pattern of increase of dissimilarity with the geographical distance was significant, though weak, in the continental shelf, but was not significant in the continental slope and SPP. The distance decay relationship of the shelf indicated that the turnover of the benthic microbial communities in this province are influenced by environmental gradients and dispersal limitation. Whereas, in the slope and SPP there is no significant relationship between geographic distance and community similarity, meaning that microbial communities of these provinces do not become more dissimilar as the distance between them increases. This lack of a pattern in the slope and SPP could indicate that spatial distance is not a primary factor influencing the assembly of these communities. Instead, other factors—such as environmental conditions, habitat connectivity, or stochastic processes—may play more substantial roles in shaping community composition (see Fig. 4).

**Figure 5:**
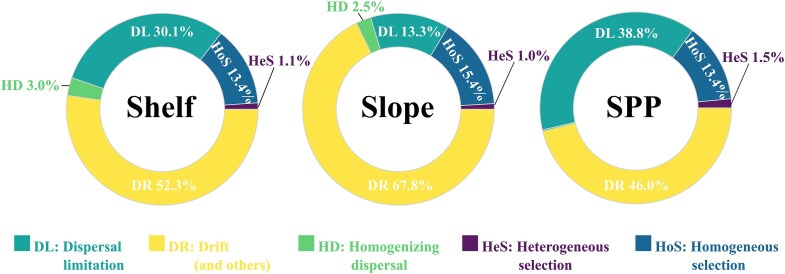
Relative importance of the ecological process in benthic communities assembly of the three physiographic provinces of the SB.

The dissimilarity as a function of the geographic distance indicated that the dispersal, selection and drift acted differently upon the assembly of microbial benthic communities across the basin. The outcome of the ecological null model analysis showed that drift was the dominant mechanism in the assembly of benthic microbial communities in the three physiographic provinces, with higher proportions of community variation explained in the continental slope (67.78%). In the continental shelf and the SPP, dispersal limitation was the second most important process (30,13% and 38.77%, respectively), whereas in the continental slope, homogeneous selection was the second most important process (15.43%) (Fig. 5). Based on the principle of the null models employed by iCAMP, the fractions of dispersal limitation, homogenizing dispersal, and drift are considered stochastic. Thus, the sum of their estimated relative importance can be used to estimate stochasticity of community assembly [18]. In the three SB provinces, the assembly of benthic communities was dominated by stochasticity, but the relative importance of the stochasticity in the continental shelf was significantly higher than in the continental slope (Cohen’s d = 2.37, *P* = .048). The drift and the dispersal limitation were significantly different between the three provinces (Cohen’s d – *P* < .05). The homogeneous selection was significantly higher in the continental slope than in the continental shelf (Cohen’s d = −2.43, *P* = .045) and the homogenizing dispersal was significantly higher in the continental shelf than in the SPP (Cohen’s d = 6.18, *P* = .0003). The NCM results aligned closely with iCAMP, suggesting a substantial influence of neutral processes in community assembly. The high variance explained (R^2^) implies high contribution of neutral processes with low influence of immigration rate (m) supporting the iCAMP results (Supplementary Table S3).

The iCAMP framework provided information on the relative importance of the different ecological processes in individual lineages (bins). For this purpose, the 29 495 benthic ASVs of the SB were divided into 1085 “bins” based on their phylogenetic relationship (ds = 0.2, bin.size.limit = 12). Our analysis showed that drift was the dominant ecological process in most bins on the continental shelf and slope, affecting 569 and 747 bins respectively. In contrast, dispersal limitation was the predominant process in the SPP, dominating 405 bins, followed by drift in 332 bins. Among the top 10 most abundant bins across the SB benthic community, five were dominated by drift in the three provinces. These include bins belonged to Nitrosopumilaceae (Bin 42), Kiloniellaceae (Bin 836 and Bin 866), Dadabacteriales (Bin 804) and Actinomarinales (Bin 911).

**Figure 6:**
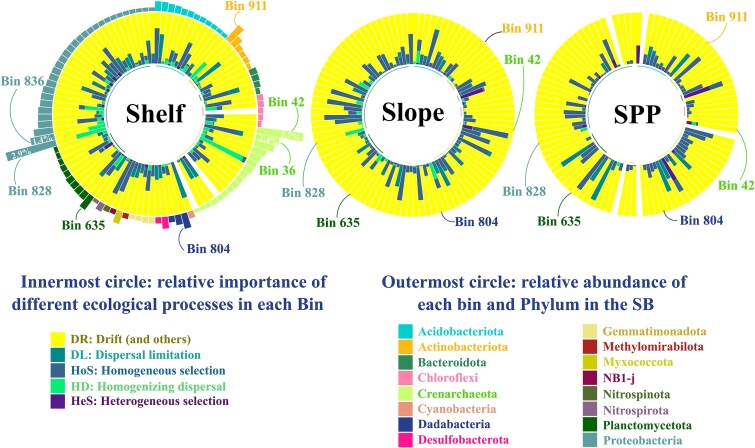
Relative importance of ecological processes across the 100 most abundant phylogenetic bins of the SB benthic microbial community. Each circular plot represents one of the three physiographic provinces; continental shelf, slope, and São Paulo Plateau (SPP), and displays two layers of information per bin. The innermost bars indicate the relative contribution of five ecological processes inferred from the iCAMP framework: drift and undominated processes (DR, yellow), dispersal limitation (DL, green), homogeneous selection (HoS, blue), homogenizing dispersal (HD, cyan), and heterogeneous selection (HeS, purple). The outermost ring shows the relative abundance of each bin, color-coded by its assigned Phylum. Highlighted bins (e.g. Bin 911, Bin 635) exemplify clades with contrasting ecological processes or abundances.

We also observed regional differences in ecological process dominance within major taxonomic groups. For example, one of the most abundant bins belonging to *Candiadatus* Nitrosopumilus (Bin 36) were dominated by homogeneous selection in the continental shelf and slope. Similarly, one of the *Pirellula*-like planctomycetes most abundant bins (Bin 635) was dominated by homogeneous selection in the SPP. Likewise, the most abundant bin overall, belonging to Hyphomicrobiaceae (Bin 828) was dominated by homogenizing dispersal in the continental shelf, and by drift in the other provinces (Fig. 6).

The ecological processes shaping the assembly of the most abundant phylogenetic bins of major microbial groups in SB showed distinct patterns across the three physiographic provinces. Most of the *Candidatus* Nitrosopumilus bins assembly were dominated by drift across all provinces, particularly at the SPP. Homogenizing selection played a significant role in some bins on the continental slope and shelf, while dispersal limitation showed limited influence. Drift also emerged as the dominant process for most *Pirellula*-like planctomycetes bins, particularly in the SPP and continental shelf. Homogenizing selection had a greater relative importance on the slope, while dispersal limitation was more influential in specific bins across the slope and SPP. Drift was also consistently the dominant process across provinces for most Woeseia bins, particularly in the SPP. Homogenizing selection had a higher influence on the slope and shelf, while dispersal limitation had varying but lower importance (Supplementary Table S4).

### Environmental factors influencing the benthic ecological processes

The influence of environmental factors was determined applying the Mantel test. We focused on the three major processes—drift, dispersal limitation and homogeneous selection- that had a higher relative importance in the assembly of the basin communities. The processes were significantly linked to sediment properties such as grain size, grain sorting (σ grain size) and mud content. However, factors related to the organic matter quality of the sediment, such as isotopic, phytopigment, and biochemical indicators also displayed high association. Drift showed the strongest correlation with the isobath (Mantel R^2^ > 0.6, *P* < .05) followed by carbohydrate concentration, δ13C, nitrogen concentration and TOC (Mantel R^2^ > 0.5, *P* < .05) on the continental shelf. In the continental slope, the mud content displayed the highest correlation with the drift (Mantel R^2^ > 0.3, *P* < .05), whereas the highest correlation with drift in the SPP was displayed with the longitude (Mantel R^2^ > 0.2, *P* < .05). Dispersal limitation showed the highest correlation with bottom water temperature and the difference in bioavailable copper concentration (ΔBio. Cu) in the continental shelf (Mantel R^2^ > 0.45, *P* < .05). In the continental slope the highest correlation with the dispersal limitation was displayed by grain sorting and isobath (Mantel R^2^ ≥ 0.4, *P* < .05). Homogeneous selection has a lower relative importance when compared to drift and dispersal limitation in the SB microbial communities. The province with the highest influence of homogeneous selection was the continental slope, which showed the strongest correlation with bioavailable aluminum variation (ΔBio. Al) (Mantel R^2^ > 0.4, *P* < .05). In the continental shelf, the homogeneous selection showed the strongest correlation with total resolved hydrocarbons (TRH) followed by coprostanol and tetradecanol concentrations (Mantel R^2^ > 0.4, *P* < .05). In the SPP the strongest correlation of homogeneous selection was showed by dotriacontanol concentration (C32:OH) (Mantel R^2^ > 0.5, *P* < .05) followed by the difference in dotriacontanol concentration (ΔC32:OH) and the difference in sediment redox potential (ΔRedox) (Mantel R^2^ > 0.4, *P* < .05) (Supplementary Fig. S16).

## Discussion

### Microbial community structure and assembly process on a regional scale

Consistent with previous marine sediment surveys, the microbial communities of SB were dominated by bacteria over archaea, with the highest bacterial dominance on the continental shelf, followed by the SPP and slope [38–40]. Gammaproteobacteria was dominant in the shelf and SPP, while Alphaproteobacteria dominated the slope. Key taxa included heterotrophic, mixotrophic, and autotrophic microorganisms involved in biogeochemical cycles [7, 41–51]. ASV richness and diversity decreased with depth, consistent with global trends [52]. The physiographic province variation in the community structure of SB surface sediment microbial communities support the existence of a biogeographic pattern and the idea that habitat is an important factor in structuring diversity. This may occur as a result of adaptation by microbial taxa to local conditions, which can be of particular importance in the rich mosaic of habitats in continental margins as SB [53–55] (see **Supplementary information** for details). Although sampling occurred over several months, we consider the influence of temporal variability on our results to be minimal, as marine sediments are relatively stable environments where microbial communities tend to exhibit limited short-term fluctuation compared to the more dynamic water column [56].

The significant influence of scale on sediment microbial community structure and assembly, suggests that comparison of the assembly processes of benthic microbial communities on a regional scale could be critical for understanding general patterns in the assembly of microbial communities in extensive ecosystems such as marine sediment [56]. Prior research on the assembly of microbial benthic communities in the ocean employed qualitative methods to determine the relative importance of ecological processes. The majority of studies, conducted in coastal areas and focused on the variation between surface and subsurface, determined that stochastic processes, such as drift and dispersal limitation, play an important role in the assembly of benthic microbial communities [57–59], as does homogeneous selection [13]. However, most of the studies have assessed the process on seashore, while the study of community assembly processes variation over space remains elusive on oceanic regional scales. Thus, our focus was to reveal the differences in microbial community assembly throughout the physiographic provinces of an oceanographic basin using a quantitative rather than a qualitative approach.

Consistent with earlier studies conducted in coastal locations, the SB displayed a stochastic-dominated assembly at the regional scale, due to the drift and dispersal limitation of high relative importance. Nonetheless, significant differences in the relative importance of the process were found between the provinces. Despite being dominant, the relative relevance of stochasticity was significantly lower in the continental slope. Dispersal limitation—another stochastic process—was the second most significant process in the continental shelf and SPP, while homogeneous selection was the second most important process in the continental slope. Benthic communities had a significantly higher relative importance of dispersal limitation in the SPP than those on the continental shelf and continental slope. From the perspective of biogeographic patterns, the relatively weak homogenous selection and dispersal limitation with or without interaction with drift, could explain the weak distance-decay relationship observed in the benthic microbial communities of the SB [60].

The dominance of ecological drift in community assembly, combined with the absence of a significant distance–decay relationship in the slope and SPP provinces, suggests a scenario in which stochastic processes shape microbial communities without strong spatial structuring. Despite this overall dominance of stochasticity, deterministic processes, particularly homogeneous selection, also played an ecologically meaningful role in community assembly. On the continental slope, homogeneous selection was the second most important process, suggesting that relatively stable environmental conditions within this province may have imposed consistent selective pressures, favoring phylogenetically related taxa. These findings may indicate relatively homogeneous environmental conditions across the slope and SPP provinces, which could contribute to broad-scale microbial connectivity and, consequently, weaken spatial turnover [61, 62]. Moreover, the absence of a distance–decay pattern may reflect the scale dependency of dispersal processes; at intermediate spatial scales, drift can override dispersal limitation [60]. With environmental conditions relatively uniform within these provinces, there are fewer selective gradients to structure community composition. Under such circumstances, community differences may arise from random demographic events, with a lesser contribution from deterministic responses to environmental variation. This can allow ecological drift to dominate community assembly without leading to a systematic increase in community dissimilarity with geographic distance. Our findings are consistent with marine studies that report a prominent influence of stochastic processes in benthic as seamounts and coastal marine sediments [63, 64]. Notably, as in our study, homogeneous selection has also been identified as a secondary but ecologically meaningful process in coastal microbial community assembly [65].

The fit of the NCM to our data, as evidenced by high R^2^ values, indicates a substantial contribution of neutral processes, particularly ecological drift, to the assembly of benthic microbial communities. The result aligns closely with those from the iCAMP framework, which also identified drift as a dominant assembly process. This result supports the view that the assembly of benthic microbial communities in the region are dominated by stochastic processes. Such drift-dominated dynamics are likely to be especially pronounced under conditions of environmental homogeneity or in physically isolated sediment patches, as previously observed in other deep-sea regions [62, 66].

The assembly mechanisms of dominant microbial groups in SB sediments revealed consistent patterns shaped by both functional traits and environmental context. *Candidatus* Nitrosopumilus, an abundant group of AOA, showed drift as the predominant process across all provinces, particularly in the SPP. This suggests that despite its specialized role in nitrogen cycling, low habitat connectivity and weak environmental gradients may enhance stochasticity. However, the influence of homogenizing selection on the slope and shelf points to a role for deterministic processes under more variable redox or ammonia conditions.

For *Pirellula*-like Planctomycetes, involved in the degradation of complex organic matter, drift also dominated, especially in the shelf and SPP. Yet, a greater influence of homogenizing selection on the slope suggests niche-based processes may become more relevant in regions with stronger environmental gradients. Likewise, *Woeseia* exhibited a similar pattern: drift predominated across provinces, but selection gained relative importance on the slope and shelf, possibly reflecting responses to spatially structured organic matter inputs.

Our results of the bin-based analyses support the hypothesis that assembly of different benthic microbial clades could be dominated by contrasting mechanisms depending on their spatial and environmental context. For the major microbial groups of the surface sediment, including *Candidatus* Nitrosopumilus, *Pirellula*-like planctomycetes, and *Woeseia* drift appeared as the primary ecological process shaping the benthic microbial communities, particularly in the deeper SPP. In contrast, homogenizing selection had a higher relative importance on the slope, a province characterized by environmental stability, where consistent conditions exert strong selective pressures that favor similar microbial communities. Dispersal limitation, while present, played a secondary role [6, 56]. These findings indicate that even highly abundant and functionally important taxa are not exempt from stochastic assembly processes, underscoring the intricate balance of ecological processes shaping microbial community assembly in marine sediments. They further highlight the importance of clade-based approaches to fully understand how selection, drift, and dispersal interact in microbial community assembly at a regional scale.

### Environmental factors mediating the balance between assembly processes in benthic microbial communities

Our focus on the surface sediment layer targets the most biologically active zone, where intense microbe–water column interactions and rapid biogeochemical cycling occur [67]. This layer is also highly responsive to nutrient and contaminant inputs, making it a key habitat for detecting spatial patterns in microbial community assembly. In this context, the environmental factors influencing community assembly were correlated with sediment characteristics, organic matter quality, and hydrochemical variables of the water mass in contact with the sediment; however, their relative influence varied across the physiographic provinces of the basin. This supports the hypothesis that key environmental drivers shaping ecological processes differ regionally. For example, in the continental slope and SPP, drift was mainly associated with physical factors such as grain size and depth. In contrast, on the continental shelf, drift correlated more strongly with organic matter properties.

Our finding that drift dominates microbial community assembly across provinces, even in regions with high microbial diversity and environmental variability, underscores the complex interplay between stochastic and deterministic processes in benthic microbial communities. The high primary production rate of the continental shelf, induced by nutrient availability derived from continental runoff and ground-water discharges, make this province a key player in global carbon storage by continental margins, the ocean areas that store over 90% of the global carbon [68]. The nutritional characteristics of these areas support a high abundance and diversity of microbial communities. Despite the high diversity, we propose that intense microbial activity, especially the rapid colonization and degradation of labile organic matter, may amplify demographic stochasticity, thereby increasing the influence of drift [38, 69]. Thus, our results suggest that the continental shelf microbial communities play a dual role: while their diversity supports efficient organic matter turnover, drift effects, likely driven by frequent population turnover and colonization dynamics, promote flexible, and responsive community structures in response to changing organic matter inputs. These findings highlight that high diversity does not exclude the dominance of stochastic processes. Instead, drift may reflect the cumulative outcome of historical contingencies, local extinctions, and demographic randomness, particularly in systems where selective pressures can rapidly change. This aligns with previous work demonstrating that even in diverse microbial ecosystems, neutral processes can dominate when environmental gradients are shallow, patchy, or temporally unstable [70, 71].

The dispersal limitation was related to sediment and bottom water properties, as well as copper variation. These findings are consistent with the fact that even in strongly connected ecosystems such as surface sediments, bottom water flow and tidal activity influence the dispersal of microorganisms and their transportation to the benthic zone [72–74]. Water temperature was one of the main environmental factors correlated with drift in the slope and dispersal limitation in the shelf. Given that conservative temperature was colinear with salinity, it is likely that salinity gradients across the physiographic provinces also influenced microbial community patterns (**Supplementary Information**). Furthermore, copper may influence dispersal limitation in benthic communities in ways distinct from other metals. While essential for biological processes, excess copper can inhibit ecosystem functions. Its unique geochemical behavior—including interactions with organic matter, redox conditions, and pH—may cause it to behave differently than other metals, thereby creating specific selective pressure [75, 76]. This demonstrates how benthic microbial communities, despite being in interconnected systems, are influenced by spatial constraints, which can impact ecological resilience and microbial services. The third most important process was homogeneous selection, which was associated with variations in aluminum, hydrocarbon and biomarkers lipids concentration, and sediment redox potential differences. These results demonstrated that a significant fraction of benthic microbial community assembly is shaped by the effect of major components of the sediment, such as aluminum [77] and organic compounds such as hydrocarbons, that are naturally widespread in marine sediments and can originate from several natural and anthropogenic sources [78]. Additionally, factors such as the origin and pathway of the organic matter, along with oceanic conditions, also play a role in influencing the distribution of lipid biomarkers and redox potential [79]. Our results provide important insights into the relationships between environmental gradients and community assembly processes, revealing consistent patterns across provinces. These findings contribute to our understanding of how environmental factors can influence microbial community assembly as inferred by the iCAMP framework. However, these spatial patterns likely interact with temporal dynamics, as seasonal and long-term changes can alter dominant taxa and assembly processes [80]. To achieve a comprehensive understanding of microbial dynamics and improve predictions of ecosystem functioning, future studies should integrate temporal variation alongside spatial analyses.

Differences in microbial community assembly processes across physiographic provinces highlight the need for province-specific management strategies to sustain key ecological services, including nutrient cycling, organic matter degradation, and carbon sequestration [81]. On the continental shelf, where microbial community assembly is dominated by drift and strongly influenced by dispersal limitation, management should prioritize reducing nutrient and contaminant inputs from coastal sources while preserving habitat connectivity [56]. Additionally, projected increases in bottom water temperature due to global warming, particularly on the continental shelf, may drive shifts in microbial assembly because rising temperatures enhance deterministic processes in sediments and amplify contaminant effects [82]. This cumulative effect underscores the urgency of implementing stricter controls on nutrient and hydrocarbon inputs since strong selection could reduce diversity and potentially destabilize ecosystem functioning. In deeper regions such as the slope and SPP, which are characterized by sediment stability and organic matter degradation rates, vulnerability to long-term disturbances such as deep-sea mining and bottom trawling is heightened. Conservation efforts should prioritize preserving habitat integrity over extended time scales. Practical measures include establishing deep-sea marine protected areas that restrict destructive activities [83]. Recognizing these spatial differences in microbial community assembly and implementing long-term environmental monitoring and impact assessments will improve predictions of ecosystem responses and support more effective marine management policies.

### Concluding remarks

This study disentangled the relative importance of ecological processes on the surface sediment microbial communities of the ocean at a regional scale. Drift, dispersal limitation and homogenous selection were the three main processes that shaped the microbial benthic communities, with variation between physiographic provinces and associated to different environmental factors by province. The balance between the ecological process is mediated by differential physicochemical factors related to the sediment characteristics and organic matter quality. The variation in key assembly environmental drivers between physiographic provinces underscores the need for province-specific approaches to understanding and managing microbial communities and their associated ecological services. For example, maintaining processes driven by hydrocarbon and lipid content on the continental shelf may require regulations on nutrient and contaminant inputs, while deeper regions, shaped by sediment characteristics and depth, would benefit from management strategies focused on long-term habitat preservation. The findings highlight the critical role of environmental factors and ecological processes in shaping sediment microbial communities, which are central to delivering key ecological services, particularly in the context of carbon cycling and storage. Our findings provide an outline for comparing benthic microbial assembly in oceanic basins and to forecast how the balance of ecological processes varied at the regional scale.

## Supplementary Material

Sup_fig1_ycaf189

Sup_fig2_ycaf189

Sup_fig3_ycaf189

Sup_fig4_ycaf189

Sup_fig5_ycaf189

Sup_fig6_ycaf189

Sup_fig7_ycaf189

Sup_fig8_ycaf189

Sup_fig9_ycaf189

Sup_fig10_ycaf189

Sup_fig11_ycaf189

Sup_fig12_ycaf189

Sup_fig13_ycaf189

Sup_fig14_ycaf189

Sup_fig15_ycaf189

Sup_fig16_ycaf189

Supplementary_info_tables_clean_ycaf189

## Data Availability

The sequencing reads generated for this study can be found in the National Center for Biotechnology Information (NCBI) database under BioProject PRJNA1219762 via the following reviewer link: https://dataview.ncbi.nlm.nih.gov/object/PRJNA1219762?reviewer=q4d8ds145pt0cqdvs52f7bimot
