# Supplementary material for: Drift, dispersal limitation, and homogeneous selection as key processes shaping prokaryotic community assembly in marine sediments
Source: ISME Commun. 2025 Oct 23;5(1):ycaf189. doi: 10.1093/ismeco/ycaf189 (PMC12619532; doi:10.1093/ismeco/ycaf189)
Supplement: Sup_fig1_ycaf189 [file sup_fig1_ycaf189.pdf]

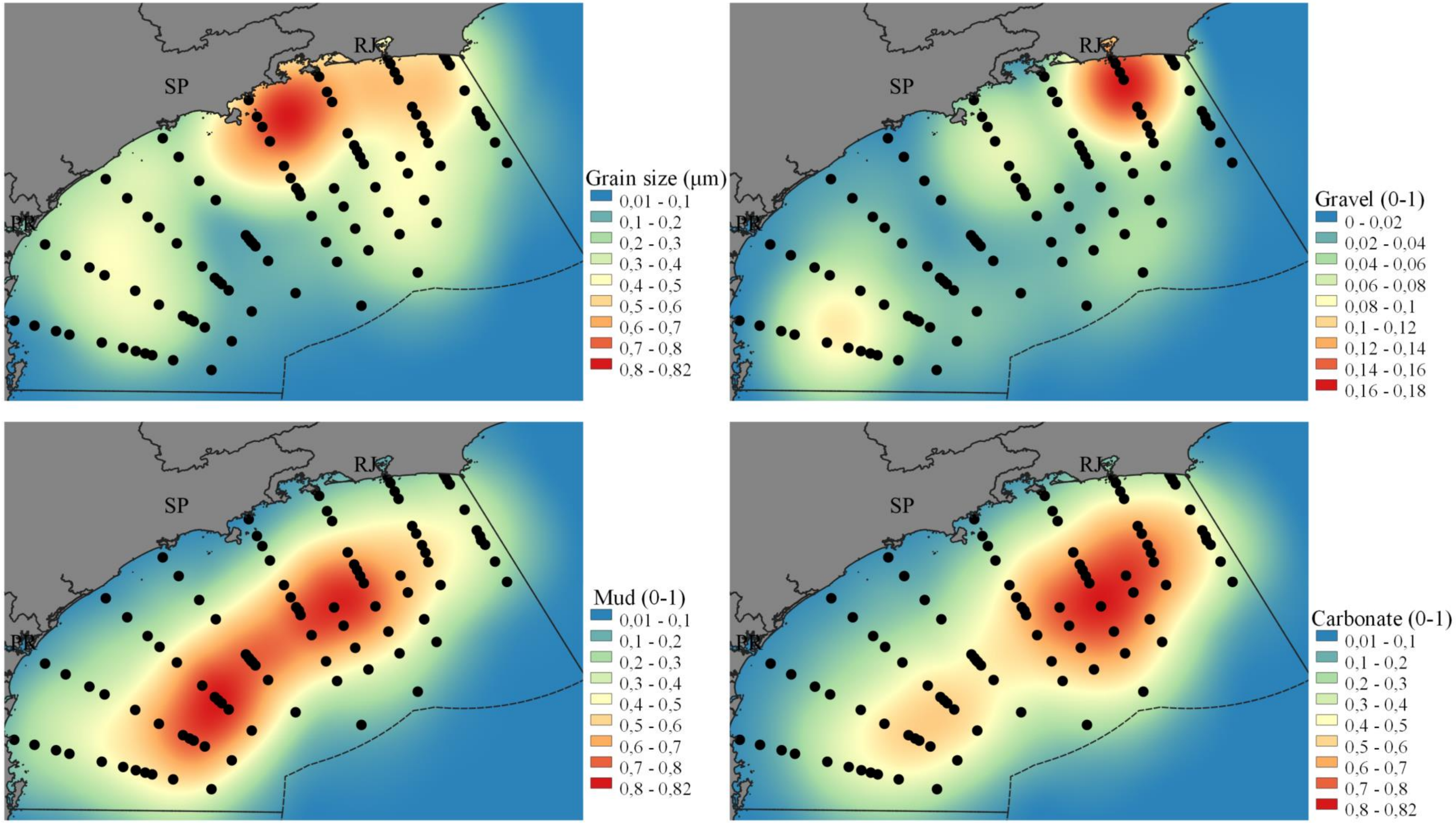

Supplementary Fig. S1. Average grain size, gravel, mud and carbonate content distribution in the surface sediment (0-2 cm) of the SB.
