# Supplementary material for: Drift, dispersal limitation, and homogeneous selection as key processes shaping prokaryotic community assembly in marine sediments
Source: ISME Commun. 2025 Oct 23;5(1):ycaf189. doi: 10.1093/ismeco/ycaf189 (PMC12619532; doi:10.1093/ismeco/ycaf189)
Supplement: Sup_fig2_ycaf189 [file sup_fig2_ycaf189.pdf]

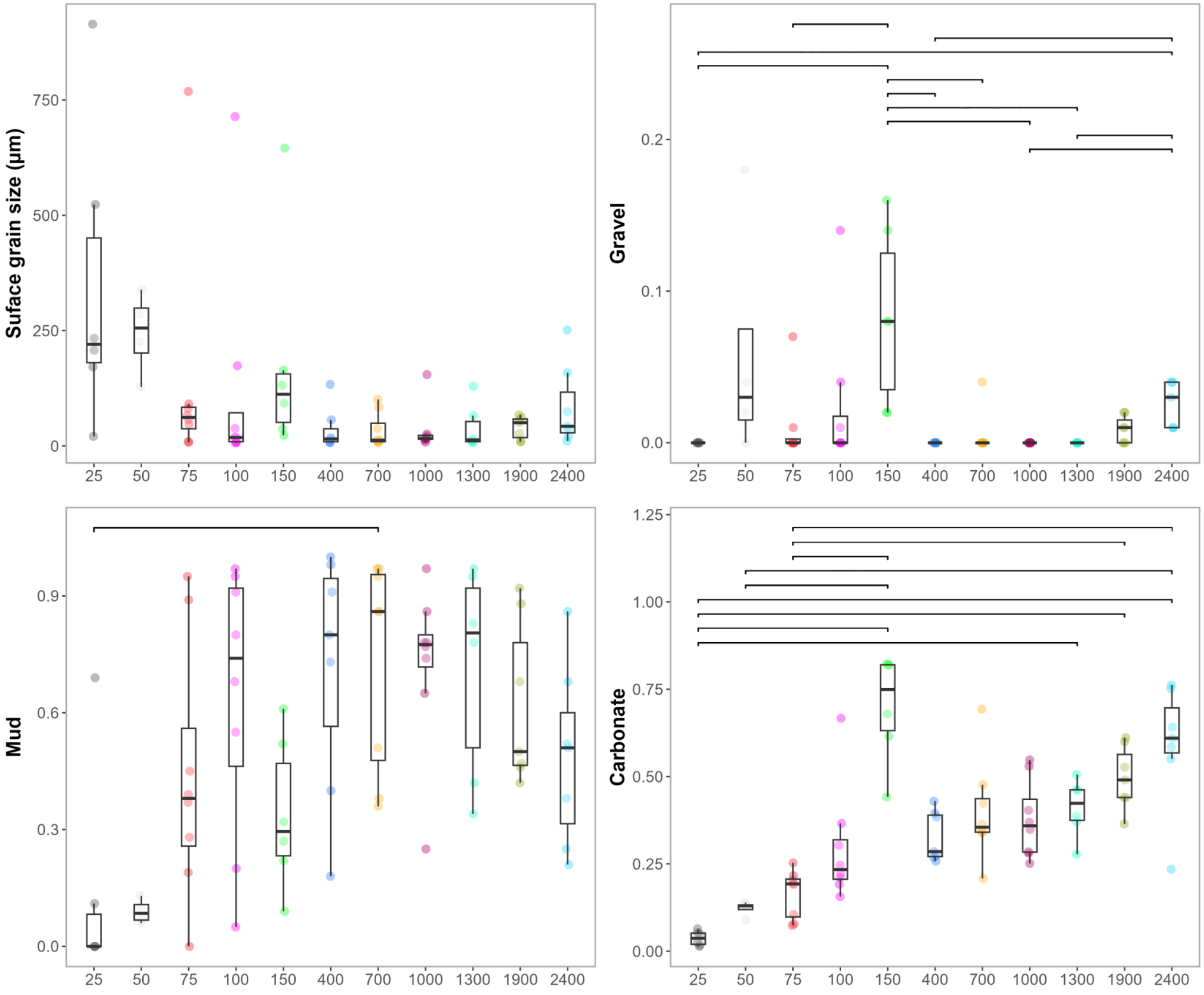

Supplementary Fig. S2. Variation by depth of sedimentological factors of the SB surface sediment: average grain size of surface sediment, gravel, mud and carbonate content (0-1) (horizontal lines show significative differences, Kruskal-Wallis p-value < 0.05).
