# Supplementary material for: Drift, dispersal limitation, and homogeneous selection as key processes shaping prokaryotic community assembly in marine sediments
Source: ISME Commun. 2025 Oct 23;5(1):ycaf189. doi: 10.1093/ismeco/ycaf189 (PMC12619532; doi:10.1093/ismeco/ycaf189)
Supplement: Sup_fig5_ycaf189 [file sup_fig5_ycaf189.pdf]

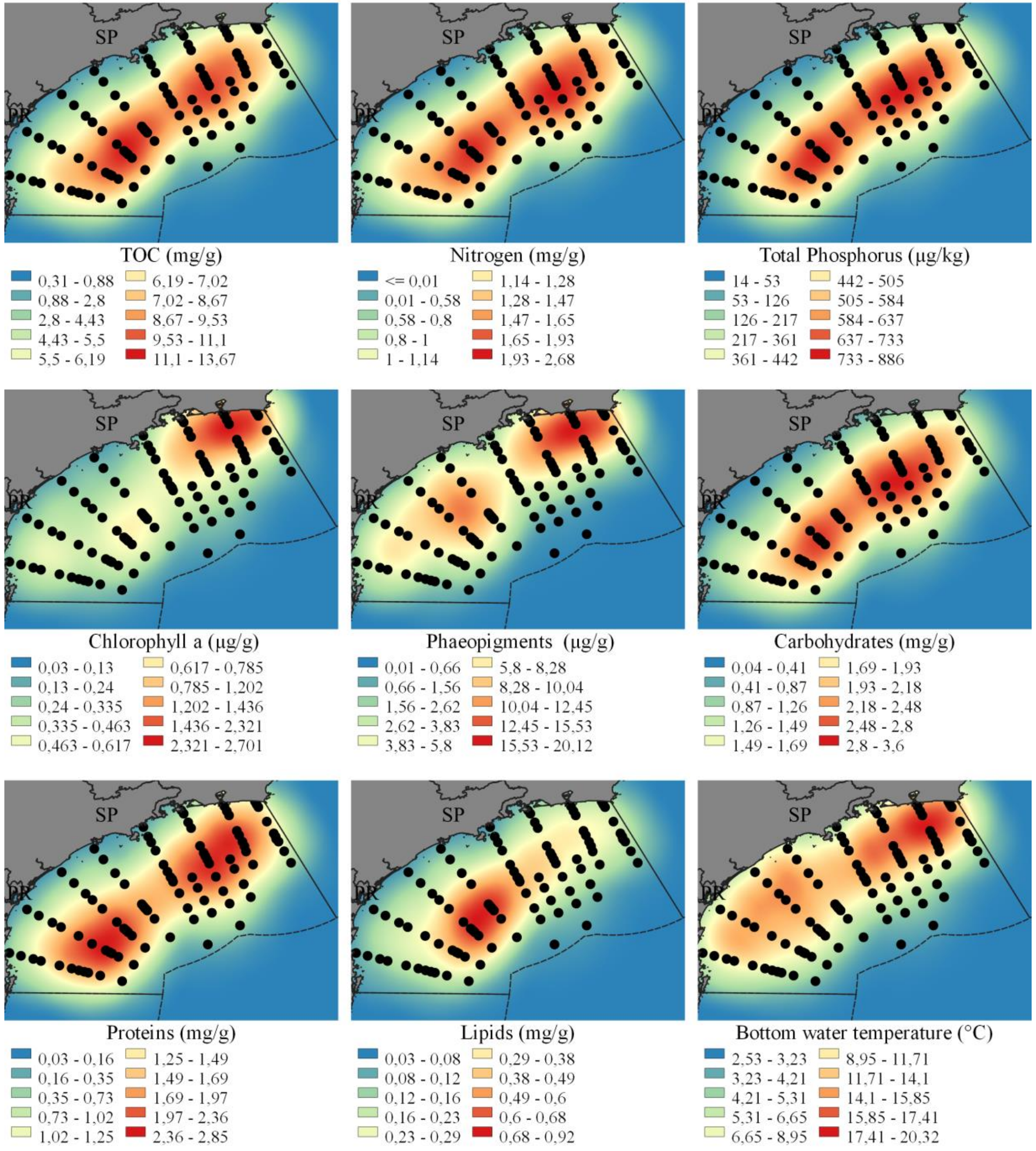

Supplementary Fig. S5. Spatial variation of elemental, phytopigment and biochemical indicators in the surface sediment, as well as bottom water conservative temperature in the SB.
