# Supplementary figures and images for: Drift, dispersal limitation, and homogeneous selection as key processes shaping prokaryotic community assembly in marine sediments
Source: ISME Commun. 2025 Oct 23;5(1):ycaf189. doi: 10.1093/ismeco/ycaf189 (PMC12619532; doi:10.1093/ismeco/ycaf189)

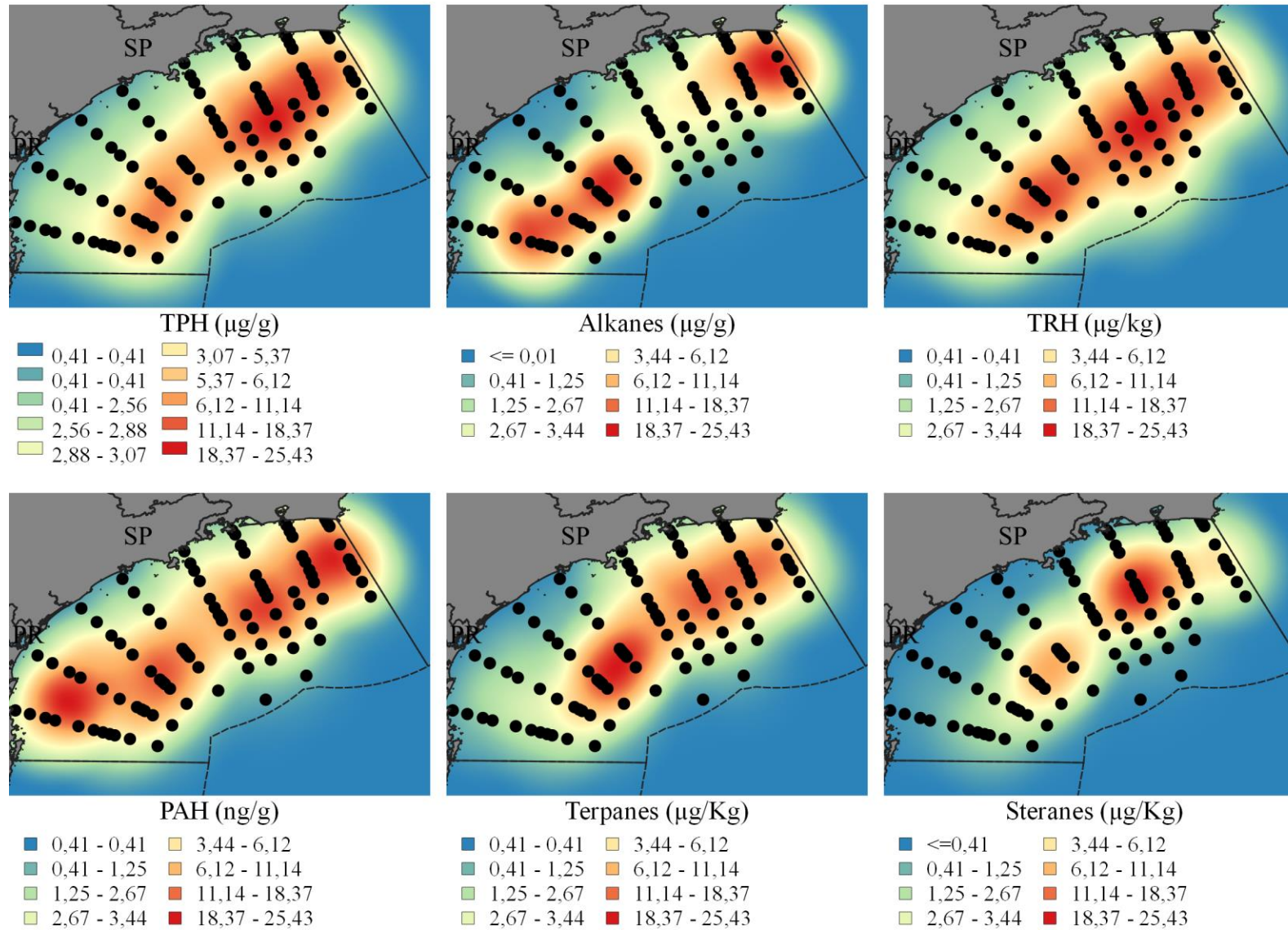

**Supplementary Fig. S7.** Spatial variation of hydrocarbons in the surface sediment of the SB.

Supplement: Sup_fig7_ycaf189 [file sup_fig7_ycaf189.pdf]
