# Supplementary material for: Drift, dispersal limitation, and homogeneous selection as key processes shaping prokaryotic community assembly in marine sediments
Source: ISME Commun. 2025 Oct 23;5(1):ycaf189. doi: 10.1093/ismeco/ycaf189 (PMC12619532; doi:10.1093/ismeco/ycaf189)
Supplement: Sup_fig9_ycaf189 [file sup_fig9_ycaf189.pdf]

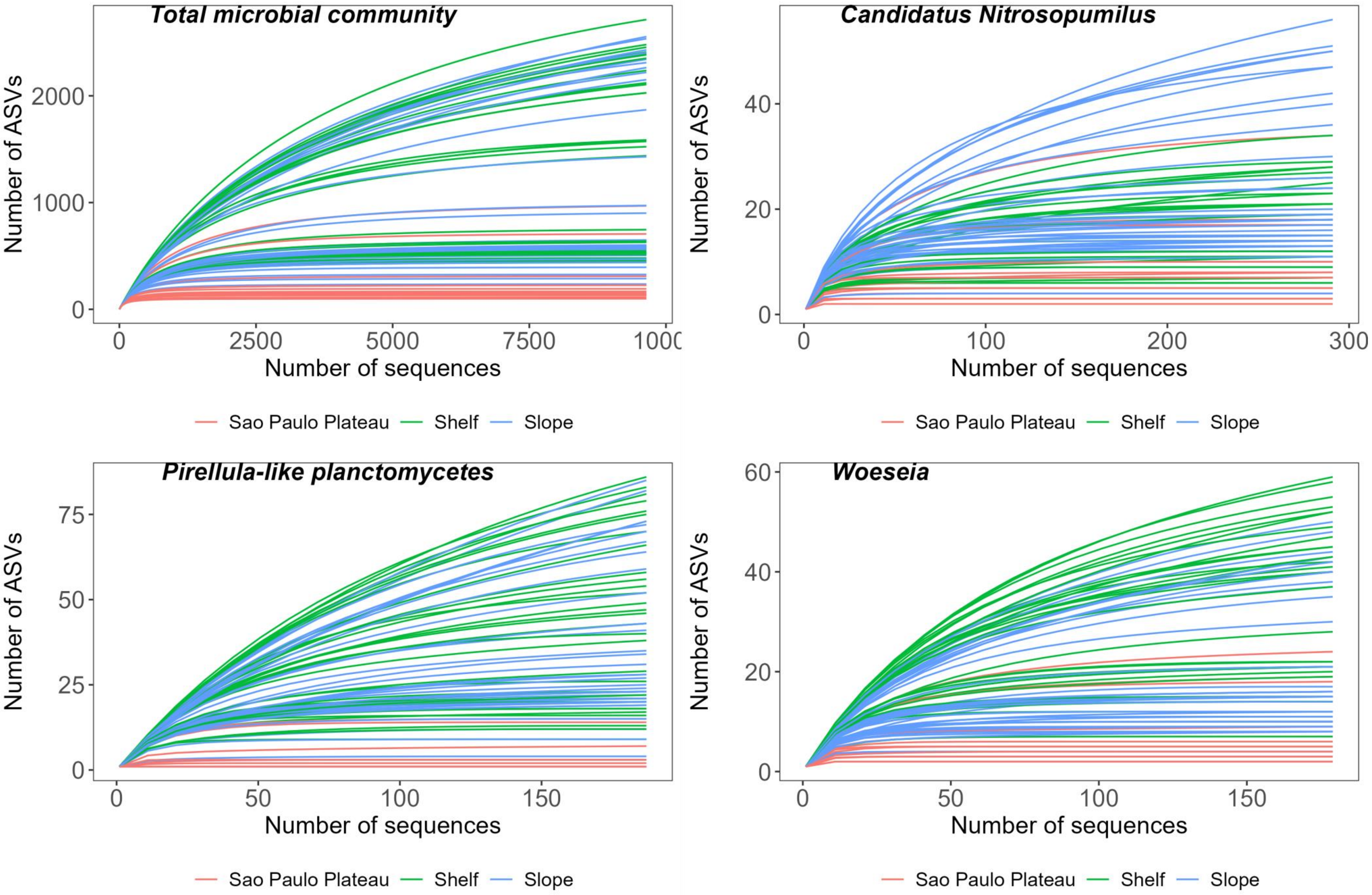

Supplementary Fig. S9. Rarefaction curves of the benthic microbial communities of the SB. Rarefied species richness (i.e., number of observed ASVs) was estimated for the total microbial community and the three major groups of benthic micro-organisms.
