# Supplementary material for: Drift, dispersal limitation, and homogeneous selection as key processes shaping prokaryotic community assembly in marine sediments
Source: ISME Commun. 2025 Oct 23;5(1):ycaf189. doi: 10.1093/ismeco/ycaf189 (PMC12619532; doi:10.1093/ismeco/ycaf189)
Supplement: Sup_fig10_ycaf189 [file sup_fig10_ycaf189.pdf]

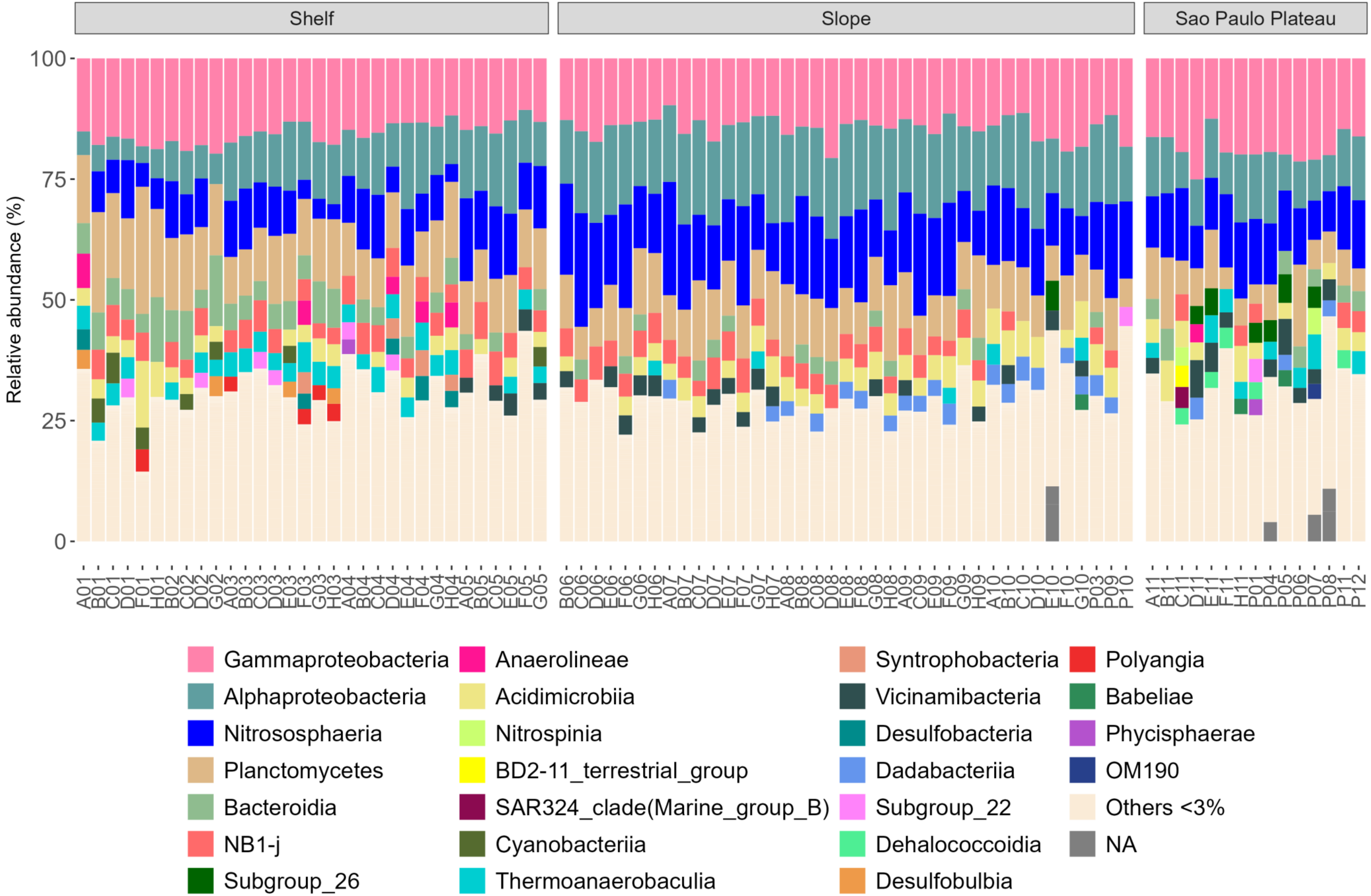

Supplementary Fig. S10. Relative abundance at Class level of the benthic microbial community over the three physiographic provinces of SB. Classes with >3% total reads are shown.
