# Supplementary material for: Drift, dispersal limitation, and homogeneous selection as key processes shaping prokaryotic community assembly in marine sediments
Source: ISME Commun. 2025 Oct 23;5(1):ycaf189. doi: 10.1093/ismeco/ycaf189 (PMC12619532; doi:10.1093/ismeco/ycaf189)
Supplement: Sup_fig11_ycaf189 [file sup_fig11_ycaf189.pdf]

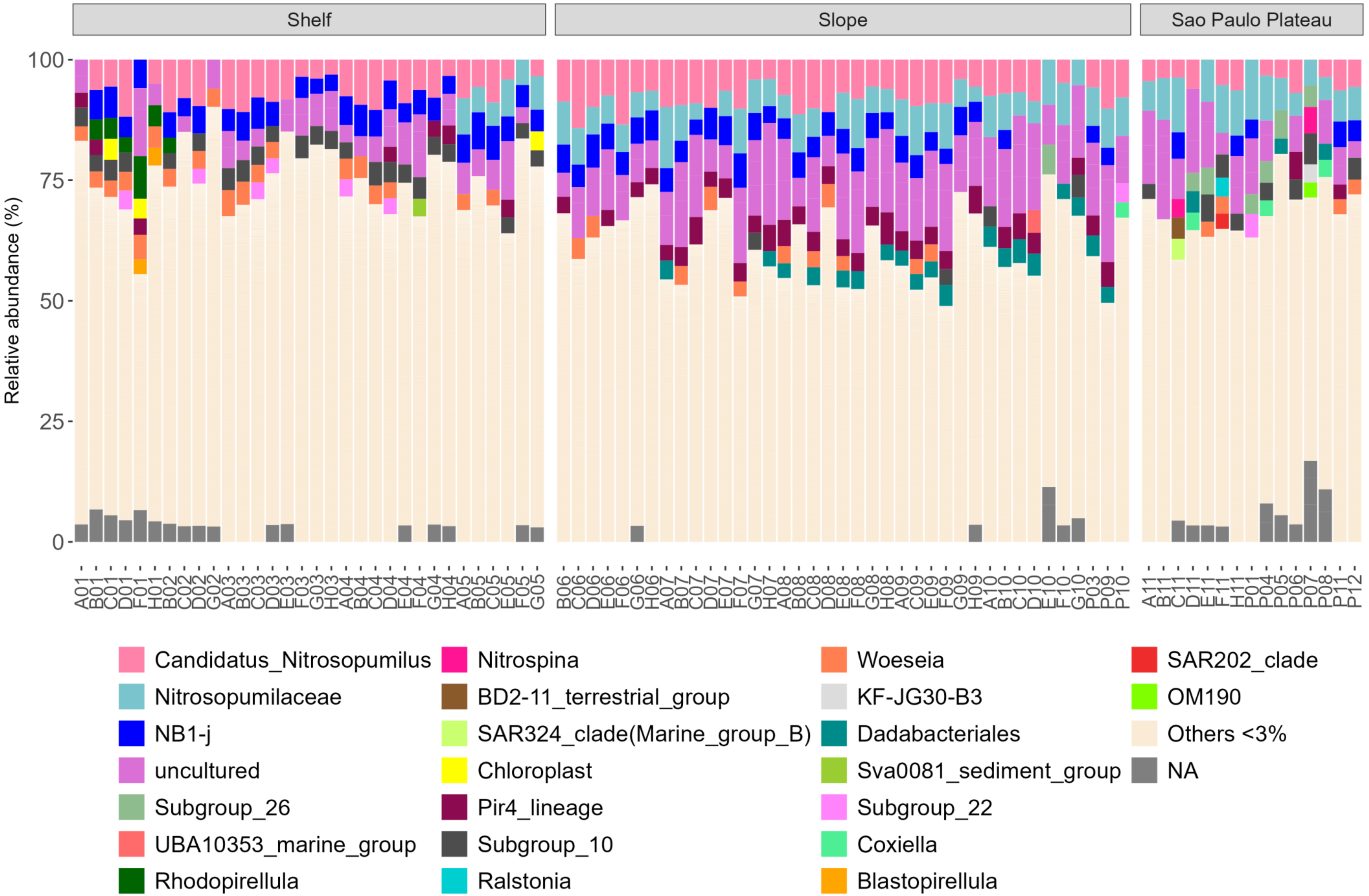

Supplementary Fig. S11. Relative abundance of *Candidatus Nitrosopumilus*, *Pirellula*-like planctomycetes (Pir4 lineage) and Woeseia, as well as other major clades of the benthic microbial community over the three physiographic provinces of SB. Classes with >3% total reads are shown.
