# Supplementary material for: Drift, dispersal limitation, and homogeneous selection as key processes shaping prokaryotic community assembly in marine sediments
Source: ISME Commun. 2025 Oct 23;5(1):ycaf189. doi: 10.1093/ismeco/ycaf189 (PMC12619532; doi:10.1093/ismeco/ycaf189)
Supplement: Sup_fig12_ycaf189 [file sup_fig12_ycaf189.pdf]

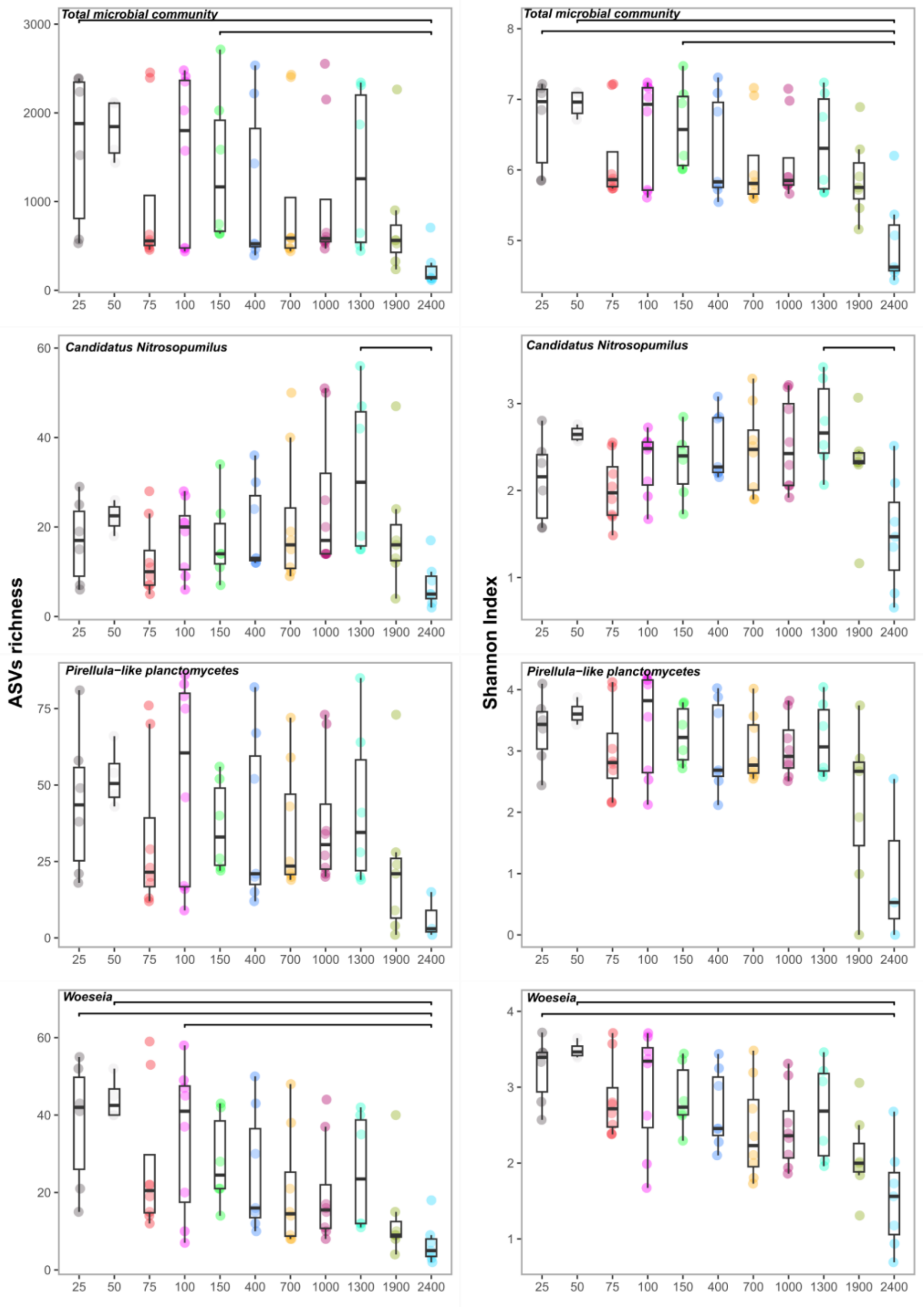

Supplementary Fig. S12. Alpha diversity of the microbial community of the SB by isobaths (horizontal lines show significative differences).
