# Supplementary material for: Drift, dispersal limitation, and homogeneous selection as key processes shaping prokaryotic community assembly in marine sediments
Source: ISME Commun. 2025 Oct 23;5(1):ycaf189. doi: 10.1093/ismeco/ycaf189 (PMC12619532; doi:10.1093/ismeco/ycaf189)
Supplement: Sup_fig13_ycaf189 [file sup_fig13_ycaf189.pdf]

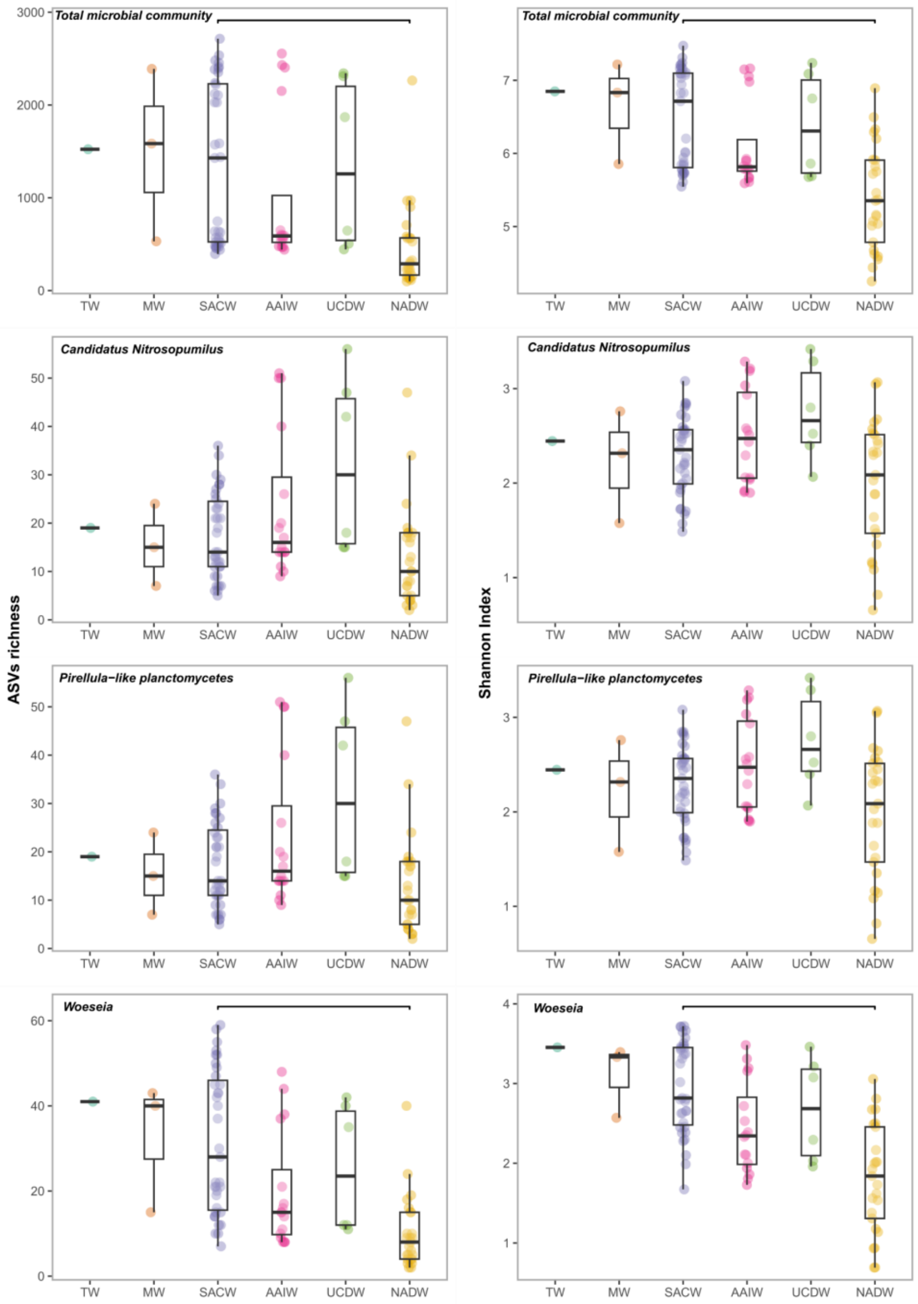

Supplementary Fig. S13. Alpha diversity of the microbial community of the SB by bottom water mass. The only station with TW in the bottom water was not considered. The bottom water masses detected in the SB where: WT (Tropical Water), MW (Mixture water), SACW (South Atlantic Central Water), AAIW (Antarctic Intermediate Water), UCDW (Upper Circumpolar Deep Water), NADW (North Atlantic Deep Water) (horizontal lines show significative differences).
