# Supplementary material for: Drift, dispersal limitation, and homogeneous selection as key processes shaping prokaryotic community assembly in marine sediments
Source: ISME Commun. 2025 Oct 23;5(1):ycaf189. doi: 10.1093/ismeco/ycaf189 (PMC12619532; doi:10.1093/ismeco/ycaf189)
Supplement: Sup_fig15_ycaf189 [file sup_fig15_ycaf189.pdf]

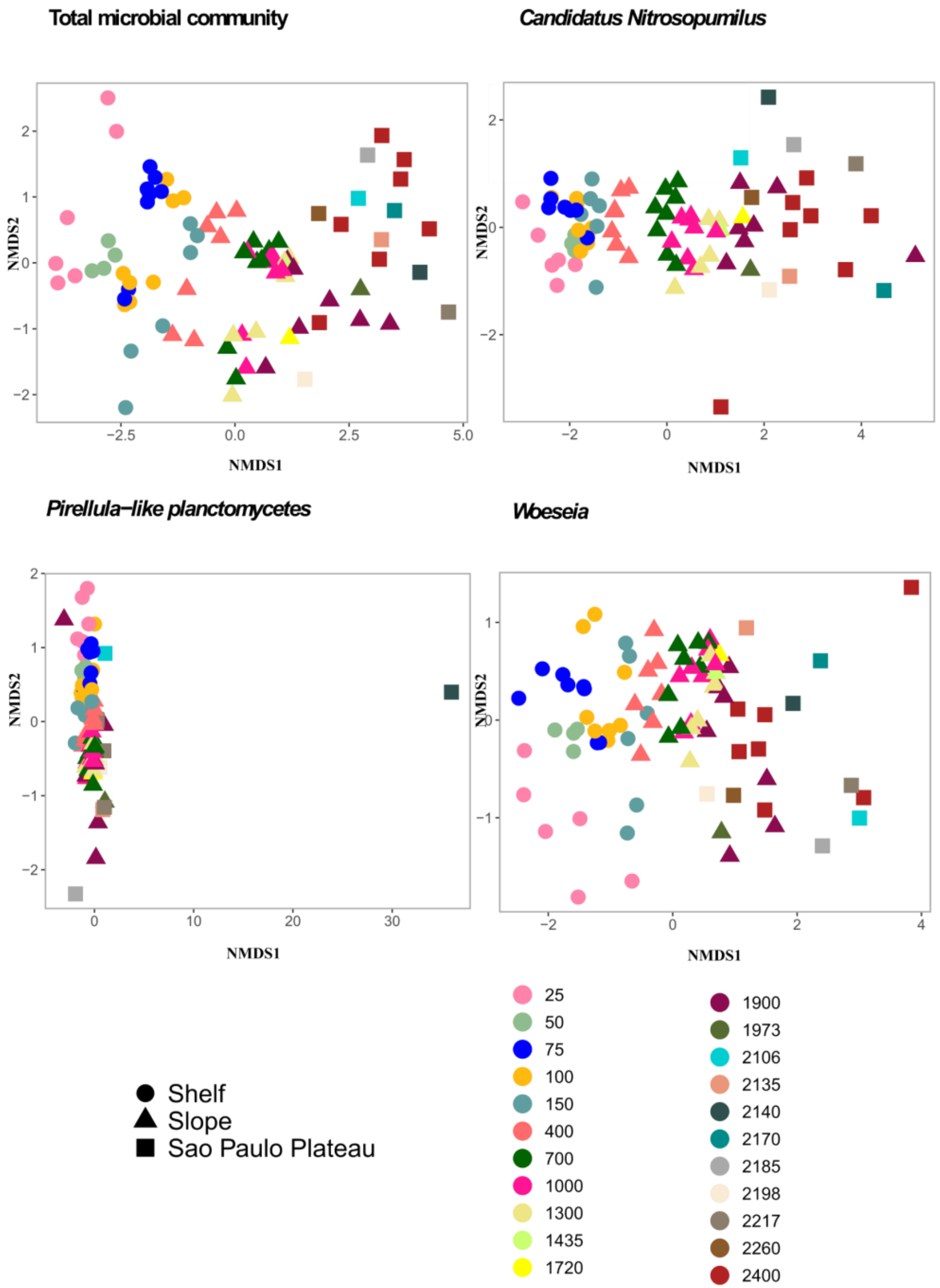

Supplementary Fig. S15. NMDS plot based on the Bray–Curtis dissimilarity of the surface sediment communities of the SB. Shapes represent the physiographic provinces of the Basin and colors represent station depth (m).
