# Supplementary material for: Drift, dispersal limitation, and homogeneous selection as key processes shaping prokaryotic community assembly in marine sediments
Source: ISME Commun. 2025 Oct 23;5(1):ycaf189. doi: 10.1093/ismeco/ycaf189 (PMC12619532; doi:10.1093/ismeco/ycaf189)
Supplement: Sup_fig16_ycaf189 [file sup_fig16_ycaf189.pdf]

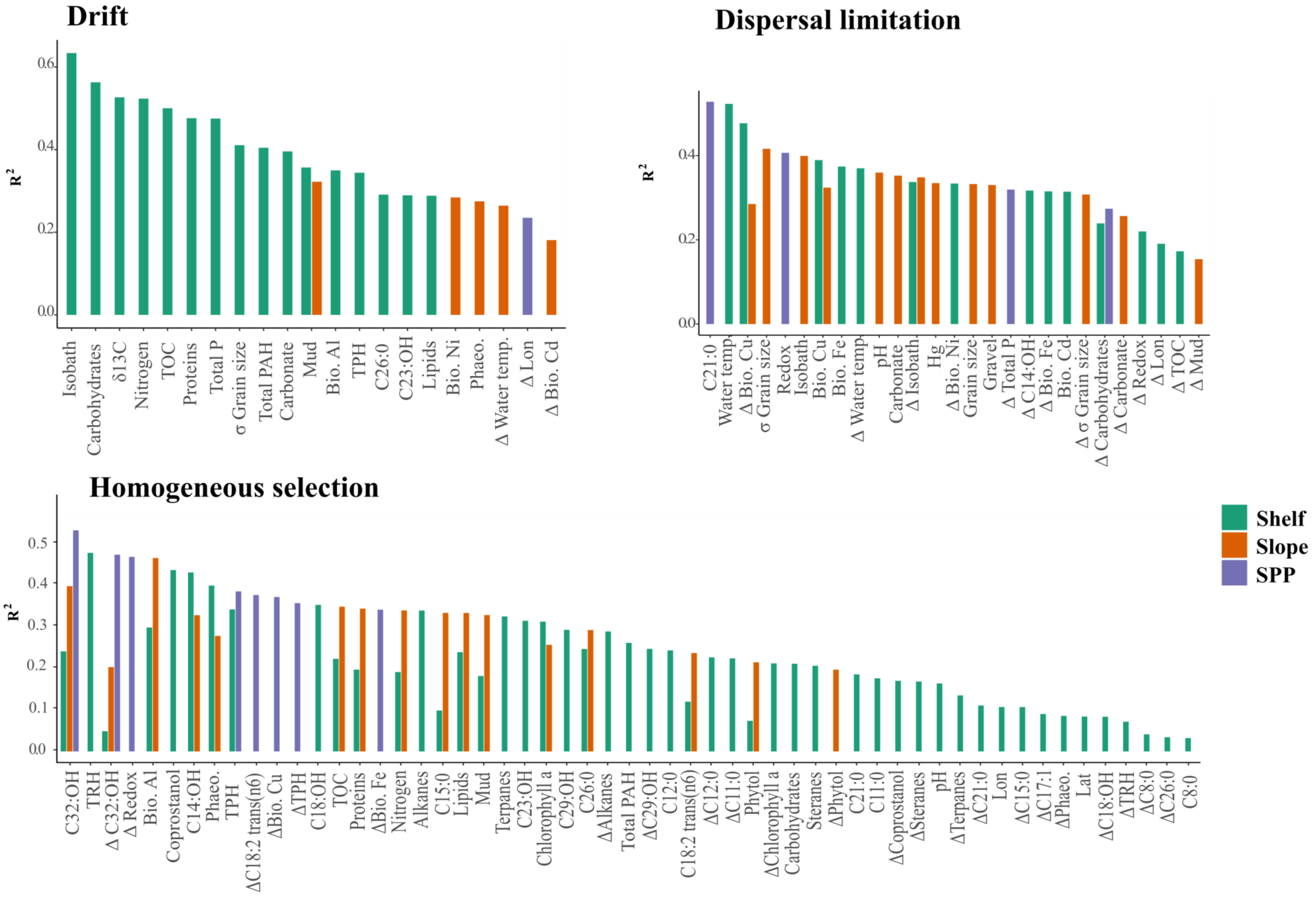

Supplementary Fig. S16. Effects of environmental factors on drift, dispersal limitation and homogeneous selection. Only significant correlations based on Mantel test were showed. The correlation was determined based on the difference (with a Δ before the name) or the mean of a factor between each pair of samples (without triangle).
