# Supplementary material for: Drift, dispersal limitation, and homogeneous selection as key processes shaping prokaryotic community assembly in marine sediments
Source: ISME Commun. 2025 Oct 23;5(1):ycaf189. doi: 10.1093/ismeco/ycaf189 (PMC12619532; doi:10.1093/ismeco/ycaf189)
Supplement: Supplementary_info_tables_clean_ycaf189 [file supplementary_info_tables_clean_ycaf189.pdf]

Supplementary information and tables- Drift, dispersal limitation and homogeneous selection as key processes shaping prokaryotic community assembly in marine sediments

Diana Carolina Duque-Castaño, Fabiana da Silva Paula, Brendan J. M. Bohannan, Alice de Moura Emilio, Julio Cezar Fornazier Moreira, Alberto G. Figueiredo Jr, Renato S. Carreira, Frederico Pereira Brandini, Daniel L. Moreira, Célio Roberto Jonck, Vivian Helena Pellizari

Supplementary Information

Selection of variables

The geological variables were standardized and were tested for normal distribution. The geological variables included: median grain size 0-2 cm, average grain size 0-2 cm, standard deviation of the grain size 0-2 cm, as well as gravel, sand, mud, clay, and carbonate content. The distribution of the geological variables, with exception of carbonate concentration, were significantly different from a normal distribution. Due to the variables distribution Spearman tested were used to test the correlation between the variables (correlation >0.70). The geological variables chose were carbonate concentration, gravel, standard deviation of the grain size 0-2 cm (colinear with no variables), mud (colinear with clay), average grain size (colinear with median grain size 0-2 cm and sand). The hydrochemical variables of the water mass in contact with the sediment variables were standardized and were tested for normal distribution. The distribution of the hydrochemical variables were significantly different from a normal distribution. Due to the variables distribution Spearman tested were used to test the correlation between the variables. The hydrochemical variables selected were conservative temperature of the water mass (colinear with salinity, dissolved oxygen and density) and mixed layer depth. The distribution of the hydrocarbonates were significantly different from a normal distribution. Due to the variables distribution Spearman tested were used to test the correlation between the variables. The variables selected were terpanes (colinear with biogeochemical markers), steranes, Total petroleum hydrocarbons- TPH (colinear with unresolved complex mixture-UCM), polycyclic aromatic hydrocarbon -PAH_T (colinear with 16_PAH), total resolved hydrocarbons-TRH and Alkanes (colinear with n_alkanes). The metal concentrations were standardized and tested for normal distribution. The distribution of the metal and metaloid concentration were significantly different from a normal distribution . Due to the variables distribution Spearman tested were used to test the correlation between the variables. The metal and metaloid concentration selected were Hg (Mercury) and the bioavailable concentrations of Al, B (that were not colinear with other metal concentrations), Cu (colinear with Ba), Fe (colinear with Zn, V, Mn, As, Pb, Cr), Cd (colinear with Zn, V, Mn, Cr and Pb) and Ni (colinear with V, As, Mn). The fatty alcohols were standardized and were tested for normal distribution (H5 were not use due to missing values). The distribution of the fatty alcohols were significantly different from a normal distribution. Due to the variables distribution Spearman tested were used to test the correlation between the variables. The fatty alcohols selected were octadecanol -C18_OH (colinear

with 28d5_22, 28d5_24_28_, 28d5, 28d0, 29d5_22, 29d5, 29d0, 30d22, C20_OH, C21_OH, C22_OH, C24_OH, C25_OH, C26_OH, C27_OH, C28_OH, C30_OH, 26d5_22, 27_d5_22, 27d5_22, 27d5), coprostanol (colinear with C24_OH, C26_OH, 26d5_22), phytol (28d5_24_28_, 28d5, 28d0), C14_OH (C16_OH, C20_OH, C22_OH and C24_OH), Tricosanol- C23_OH (C20_OH, C21_OH, C22_OH), nonacosanol C29_OH (C26_OH, C28_OH, C30_OH), C32_OH (colinear with C28_OH, 28d5_22, 28d5,28d0, 29d5_22, 29d5, 29d0, 30d22). Two of the fatty acid concentrations had not variation along all the basin, for that reason were excluded of the analysis (cis-8,11,14-eicosatrienoic acid - C20:3n6 and cis-11,14,17-eicosatrienoic acid- C20:3n3). The distribution of the fatty acids were significantly different from a normal distribution. Due to the variables distribution Spearman tested were used to test the correlation between the variables. The fatty acids selected were caprylic acid-C8_0, undecanoic acid-C11_0, cis-10-heptadecanoic acid- C17_1, linolelaidic acid- C18_2 trans_n6, heneicosanoic acid-C21_0 (that was not colinear with other fatty acids), lauric acid-C12_0, (colinear with C20_4n6_ARA, C18_2cis_n6, C18_0, C18_1 trans_n9, C18_1 cis_n9), pentadecanoic acid (colinear with C13_0, C14_0, C14_1, C15_1, C16_0, C16_1, C17_0), cerotic acid-C26_0 (colinear with arachidonic acid- C20_0, behenic acid- C22_0, lignoceric acid-C24_0, montanic acid-C28_0). As too much lipidic variables were selected the collinearity between biological macromolecules and the selected fatty alcohols and fatty acids were tested. The distribution of lipids were significantly different from a normal distribution. Due to the variables distribution Spearman tested were used to test the correlation between the variables. Just the biopolymeric carbon were excluded because it was colinear with carbohydrate and protein concentration. The final 48 selected variables were latitude, longitude, isobath, sediment redox potential, sediment pH, total organic carbon (TOC) concentration, Nitrogen concentration, total Phosphorus concentration, chlorophyll-*a* concentration, phaeopigments concentration, $\delta^{13}C$, average sediment particle size, standard deviation of granulometry, gravel content, mud content, carbonate content, conservative temperature of the near bottom water, total petroleum hydrocarbons, alkanes, total resolved hydrocarbons, polycyclic aromatic hydrocarbon, terpanes, steranes, the concentration of bioavailable elements Aluminum, Boron, Cadmium, Copper, Iron and Nickel, total Mercury, carbohydrates concentration, protein concentration, lipid concentration, myristic acid ($C_{14}OH$), octadecanol ($C_{18}OH$), Fitol ($C_{20}H_{40}O$), Tricosanol ($C_{23}OH$), nonacosanol ($C_{29}OH$), 1-Dotriacontanol ($C_{32}OH$), Coprostanol ($C_{27}H_{48}O$), caprylic acid ($C_8H_{16}O_2$), undecanoic acid ($C_{11}H_{22}O_2$), lauric acid ($C_{12}H_{24}O_2$), pentadecanoic acid ($C_{15}H_{30}O_2$), cis-10-heptadecanoic acid ($C_{17}H_{32}O_2$), linolelaidic acid ($C_{18}H_{32}O_2$), heneicosanoico acid ($C_{21}H_{42}O_2$) and cerotic acid ($C_{25}H_{51}COOH$).

Environmental characteristics of physiographic provinces

The average surface grain size displayed no significant changes between isobaths. Whereas, when compared to slope and SPP isobaths, the continental shelf gravel content was significantly higher. Surface sediments had a higher mud content in the slope and SPP, with significantly higher mud content in the 700 m isobath of the slope compared to the 25 m isobath of the shelf. Carbonate content increased significantly from the inner to

the outer shelf, as well as from the higher to the lower slope and SPP. The highest carbonate content was observed in sediments from the outer shelf and shelf-break. Concentration increased over the slope towards the deep ocean (**Supplementary Fig. S1 and S2**). Other physicochemical factors such as redox potential, pH, metal and metalloid concentration (total mercury and bioavailable aluminum, boron, cadmium, copper, iron and nickel) varied within the basin. The redox potential ranged from -212 to 296, with negative values on the shelf and positive values in the slope and SPP. The sediment pH varied between 6.85 and 8.0, with more neutral values on the shelf and higher alkalinity in the slope and SPP. The redox, sediment pH as well as the bioavailable Cu and total Hg levels all increased with depth, with platform isobaths having significantly lower values than slope and SPP isobaths. Bioavailable B and Fe concentrations tended to decrease with depth, but there were no significant differences between isobaths. Similarly, no significant differences in bioavailable Cd and Ni concentrations were found between isobaths in the basin. (**Supplementary Fig. S3 and S4**). The physiographic provinces and depths showed differences in the organic matter quality of the sediment determined by elemental, isotopic, phytopigment, and biochemical indicators. The TOC concentration ranged from 0.3 to 13.7 mg/g in the surface sediment. The total organic carbon (TOC) concentrations were generally higher along most slope isobaths compared to the shelf, particularly at depths of 700, 1000, and 1300 m. However, the 75 m and 100 m isobaths on the shelf also exhibited high TOC concentrations, comparable to those on the slope. In contrast, TOC levels in the SPP were low, similar to those observed in the 150 m and 400 m isobaths of the shelf. The total nitrogen concentration ranged between 0.01 and 2.68 mg/g and followed the spatial variation of TOC, indicating a common source [1]. The biopolymeric compounds, which comprised the total concentrations of carbohydrates, proteins, and lipids, followed a similar bathymetric gradient as TOC. The phytopigments chlorophyll-*a* and phaeopigments, on the other hand, had the maximum concentrations in the middle shelf, at 75m and 100m surface sediments. Furthermore, the bottom water conservative temperature significantly decreased with depth along the different provinces (**Supplementary Fig. S5 and S6**). Additionally, the total petroleum hydrocarbons (TPH) concentrations ranged from 2.31 to 23.35 μg/g and followed a similar pattern to TOC, with increasing concentrations in the shelf towards the 75 m and 100 m isobaths and the highest concentrations in the slope at 1000 m and 1300 m. The 400 m isobath had the highest amounts of alkanes, whereas the 75 m and 100 m isobaths had larger concentrations of polycyclic aromatic hydrocarbons. Terpane concentrations increased in the shelf toward the mild shelf (75 m and 100 m isobaths), but were highest in the slope at 1900 m. The total resolved hydrocarbons (TRH) tended to rise with depth across the provinces and steranes showed the highest concentrations on the slope, at 700m and 1300m isobaths (**Supplementary Fig. S7 and S8**).

Microbial diversity

Bacteria dominated the surface sediment microbial community in the SB. After rarefaction, they represented 87.3% of the total reads compared to 12.7% of archaeal reads. Three bacterial classes accounted for 41% of all reads: Gammaproteobacteria (16% of all reads), Alphaproteobacteria (13.5%), and Planctomycetes (11.5%). The

Nitrososphaeria class comprised the majority of archaeal reads (97.3%). Twelve other classes contributed more than 1% for the total reads: NB1-j (4.41%), Acidimicrobiia (4.24%), Bacteroidia (3.84%), Thermoanaerobaculia (3.29%), Vicinamibacteria (2.84%), Dadabacteriia (1.97%), Anaerolineae (1.89%), Subgroup_22 (1.72%), BD2-11_terrestrial_group (1.24), Phycisphaerae (1.20 %), Dehalococcoidia (1.10%) and Polyangia (1.07 %). Woeseia (20.2% of the Gammaproteobacteria reads) and an uncultured bacterium of the AT-s2-59 order (10.8% of the Gammaproteobacteria reads) were the most abundant Gammaproteobacteria. The Alphaproteobacteria were dominated by the Families Kiloniellaceae, Hyphomicrobiaceae and Methyloligellaceae (together 73.4% of the Alphaproteobacteria reads), while Planctomycetes was dominated by Pirellulaceae (88.9% of the Planctomycetes). **Supplementary Fig. S10** shows the relative abundance of the Classes with more than 1% of total 16S rRNA reads. The continental shelf exhibited the highest abundance of bacterial reads and the lowest abundance of archaeal reads whereas the slope had the highest abundance of archaeal reads between the provincesThe best-represented microbial groups were identified in each physiographic province of the SB. Bacteria accounted for 90.6% of the province reads, while Archaea accounted for 9.37%. On the slope Bacteria represented 84.5% of the province reads and Archaea 15.5% of the province reads. In the SPP Bacteria represented 87.5 % of the province reads and Archaea 12.5%. Variation in the most abundant Classes by region was also analyzed. Gammaproteobacteria was the most abundant Class in the continental shelf and the SPP, whereas Alphaproteobacteria was the most abundant Class in the slope. The archaeal Class Nitrososphaeria was the second most abundant prokaryotic class in the slope and the SPP, and the fourth most abundant in the continental shelf. Planctomycetes, Bacteroidia, Acidimicrobiia, NB1-j, Thermoanaerobaculia and Vicinamibacteria were consistently in the top ten most abundant Classes in the three provinces.

The isobath analysis showed that for the microbial community and *Woeseia* the 25 m and 150 m isobaths had significantly higher richness than the 2400 m isobath (Kruskall-Wallis, chi-squared = 20.05, p-value = 0.03 and chi-squared = 29.41, p-value = 1.07 e-3, respectively). However, for *Candidatus* Nitrosopumilus and *Pirellula*-like planctomycetes there were no significantly richness differences between most of the isobaths, with the exception of a significantly higher richness of *Candidatus* Nitrosopumilus in 1300 m isobath when compared with the 2400 m (Kruskall-Wallis, chi-squared = 20.45, p-value = 0.03). In a similar way, the Shannon index showed significantly higher diversity of continental shelf isobaths with the SPP isobath of 2400m, no difference in the diversity of *Pirellula*-like planctomycetes, and the highest diversity of *Candidatus* Nitrosopumilus at 1300 m (**Supplementary Figure S12**). The bottom water mass showed little significant influence on alpha diversity, except for the total microbial community and *Woeseia* in stations with SACW on the continental shelf and superior continental slope, that showed significantly higher diversity than stations where the bottom was dominated by NADW, which correspond to SPP stations (**Supplementary Figure S13).** The α-diversity measures showed no significant differences between the transects for the total microbial community as well as for *Woeseia*, a significant difference between the transects F and G, in the northern extreme of the SB was observed for *Candidatus* Nitrosopumilus and *Pirellula*-like planctomycetes (**Supplementary Figure S14**). Richness and diversity were generally higher in shallow continental shelf isobaths

compared to deeper SPP isobaths, particularly for the total microbial community and *Woeseia*. *Candidatus* Nitrosopumilus exhibited peak diversity at intermediate depths of the slope (1300 m), while Pirellula-like planctomycetes showed no differences across isobaths. Bottom water masses also influenced diversity, with higher values in stations under SACW compared to NADW. Lastly, differences in diversity were observed between northern transects for specific microbial groups. Variation in organic matter quality were associated with a decrease in the richness and abundance of marine oxic sediment microbial communities with depth, particularly bacterial communities, with continental margin environments exhibiting more diversity than sediments at deeper portions of the ocean [2]. The weak distance-decay pattern observed in the slope and SPP communities could be influenced by the interplay of homogeneous selection and dispersal limitation, with or without the influence of drift, highlighting the complexity inherent in elucidating the processes driving biogeographic patterns [3, 4].

References

1. Carreira RS, Lazzari L, Ceccopieri M, Rozo L, Martins D, Fonseca G, et al. Sedimentary organic matter accumulation provinces in the Santos Basin, SW Atlantic: insights from multiple bulk proxies. *Ocean Coast Res* 2023;**71**:e23030. https://doi.org/10.1590/2675-2824071.22061rsc
2. Hoshino T, Doi H, Uramoto G-I, Wörmer L, Adhikari RR, Xiao N, et al. Global diversity of microbial communities in marine sediment. *Proceedings of the National Academy of Sciences* 2020;**117**:27587–27597. https://doi.org/10.1073/pnas.1919139117
3. Zhou J, Ning D. Stochastic Community Assembly: Does It Matter in Microbial Ecology? *Microbiol Mol Biol Rev* 2017;**81**:e00002-17. https://doi.org/10.1128/MMBR.00002-17
4. Wang K, Yan H, Peng X, Hu H, Zhang H, Hou D, et al. Community assembly of bacteria and archaea in coastal waters governed by contrasting mechanisms: A seasonal perspective. *Molecular Ecology* 2020;**29**:3762–3776. https://doi.org/10.1111/mec.15600

Supplementary Tables

Supplementary Table S1. Sequence, ASVs and alpha diversity information of samples.

	Size	Seqs per sample	Total ASVs	Shannon
**Total microbial community**	86	9642	29495	8.442521
***Candidatus* Nitrosopumilus**	86	291	273	4.083803
***Pirellula*-like planctomycetes**	80	187	790	4.979722
Woeseia	86	179	452	4.773308

Supplementary Table S2. Most abundant Classes of surface sediment microorganism by physiographic province in the SB.

	Continental shelf*	Continental slope*	SPP*
**1**	Gammaproteobacteria (15.7)	Alphaproteobacteria (16.9)	Gammaproteobacteria (19.6)
**2**	Planctomycetes (13.8)	Nitrososphaeria (15.8)	Nitrososphaeria (12.7)
**3**	Alphaproteobacteria (10.5)	Gammaproteobacteria (14.7)	Alphaproteobacteria (11.2)
**4**	Nitrososphaeria (9.20)	Planctomycetes (11.0)	Planctomycetes (8.20)
**5**	Bacteroidia (5.50)	Acidimicrobiia (4.71)	Acidimicrobiia (4.07)
**6**	NB1-j (4.97)	NB1-j (4.59)	Thermoanaerobaculia (3.68)
**7**	Thermoanaerobaculia (4.06)	Vicinamibacteria (3.13)	Vicinamibacteria (3.54)
**8**	Acidimicrobiia (3.75)	Dadabacteriia (2.95)	Bacteroidia (2.81)
**9**	Anaerolineae (2.35)	Bacteroidia (2.87)	NB1-j (2.78)
**10**	Vicinamibacteria (2.17)	Thermoanaerobaculia (2.52)	Subgroup_26 (2.70)

*The numbers in parentheses indicate the relative abundance (%) in each province reads

Supplementary Table S3. Fitting of the total microbial community to the neutral community model (NCM). Rarefied ASVs abundance table of a community assembly was used to fit the NCM. The samples were divided by physiographic province. R^2 is the overall fitness to the NCM, which represents the contribution of neutral processes. Low immigration rate (m) indicates low species dispersal in the community assembly (i.e., high dispersal limitation)

Physiographic province	R2	m
**Continental shelf**	0.6863602	0.06083579
**Continental slope**	0.7183841	0.03822484
**SPP**	0.2434947	0.004202595

Supplementary Table S4. Relative importance of the ecological process in the assembly of the phylogenetic bins of major microbial groups of the SB

		Continental shelf					Continental slope					SPP				
		HeS	HoS	DL	HD	DR	HeS	HoS	DL	HD	DR	HeS	HoS	DL	HD	DR
***Ca.* Nitrosopumilus**	Bin35	0.62	5.98	2.44	11.11	79.85*	1.05	35.87	0.92	0.62	61.54*	1.07	55.56*	0.00	0.00	43.37
	Bin36	0.00	67.25*	10.06	0.06	22.64	19.73	70.01*	0.19	1.41	8.66	0.00	0.00	0.00	0.00	0.00
	Bin37	0.00	38.79*	38.07	0.00	23.14	0.00	28.68	0.00	0.00	71.32*	0.00	0.00	0.00	0.00	100.00*
	Bin38	0.00	0.00	0.00	0.00	100.00*	0.00	2.47	51.29*	0.01	46.22	0.00	1.00	9.22	0.00	89.78*
	Bin39	0.00	0.00	0.00	0.00	0.00	0.00	3.48	21.48	0.00	75.04*	0.00	3.08	60.95*	0.00	35.97
	Bin40	0.00	6.45	0.00	93.55*	0.00	2.08	63.41*	6.79	0.03	27.69	0.00	23.94	0.00	0.00	76.06*
	Bin41	0.00	19.04	0.00	5.88	75.08*	0.43	17.97	19.75	0.00	61.85*	5.54	2.74	42.21	0.00	49.50*
	Bin42	0.00	0.23	2.43	15.36	81.98*	0.27	0.43	2.85	2.99	93.47*	10.13	4.16	0.00	8.36	77.35*
**Pir4_lineage**	Bin636	0.00	12.67	3.19	0.00	84.14*	0.00	37.19	0.60	0.13	62.08*	0.00	7.14	0.00	0.00	92.86*
	Bin638	0.00	8.60	56.81*	0.00	34.59	2.42	0.83	30.39	0.00	66.36*	0.00	0.00	0.00	0.00	100.00*
	Bin639	0.00	8.42	44.80*	0.00	46.78	0.00	5.56	61.36*	0.00	33.08	0.00	0.00	28.57	0.00	71.43*
	Bin640	2.85	53.16*	15.14	0.00	28.85	3.89	0.00	0.00	1.03	95.08*	0.00	0.00	0.00	0.00	0.00
	Bin648	0.00	2.25	0.00	0.92	96.83*	0.00	5.19	0.26	0.08	94.48*	0.00	35.71	0.00	0.00	64.29*
	Bin649	0.45	0.76	47.41	0.00	51.38*	0.00	2.96	37.46	0.00	59.58*	0.00	0.00	0.00	0.00	0.00
	Bin652	7.54	7.72	0.00	2.17	82.57*	4.89	33.18	24.59	0.00	37.34*	0.00	5.03	8.63	0.00	86.34*
	Bin653	0.34	8.24	0.00	3.66	87.76*	0.00	5.77	0.11	0.00	94.12	0.00	4.68	5.63	0.00	89.69
	Bin656	1.12	5.53	71.36*	0.00	21.99	0.00	2.62	16.00	0.00	81.38	0.00	0.00	0.00	0.00	0.00
	Bin657	26.31	13.25	15.77	0.56	44.11*	0.00	3.45	14.97	0.00	81.58	0.00	0.00	0.00	0.00	0.00
Woeseia	Bin1036	0.26	25.14	2.03	1.13	71.44*	0.82	65.43*	0.00	0.79	32.96	0.00	19.35	0.00	0.00	80.65*
	Bin1037	3.32	2.13	72.15*	0.00	22.39	0.00	3.28	43.18	0.00	53.54*	0.00	0.00	0.00	0.00	0.00
	Bin1038	0.89	2.83	0.00	0.10	96.18*	8.82	6.30	0.00	0.00	84.88*	0.00	27.20	5.56	0.00	67.24*
	Bin1039	0.00	21.63	3.30	0.00	75.07*	0.00	16.60	27.00	0.00	56.40*	2.66	0.00	3.57	0.00	93.77*
	Bin1040	8.67	22.58	0.63	1.08	67.04*	0.59	29.91	1.77	0.28	67.45*	0.00	3.06	0.00	0.00	96.94*
	Bin1041	0.00	12.13	42.17	0.00	45.70*	2.44	4.09	21.83	0.00	71.64*	0.00	2.45	68.12*	0.00	29.42
	Bin1042	0.00	8.84	41.67	0.00	49.50*	2.25	7.07	38.71	0.11	51.87*	0.00	5.19	0.00	0.00	94.81*
	Bin1043	0.00	3.44	0.00	0.43	96.13*	0.47	3.67	48.17	0.00	47.68*	0.00	4.15	66.64*	0.00	29.20
	Bin1044	0.00	2.78	68.56*	0.00	28.65	0.00	0.00	0.00	4.74	95.26*	0.00	0.00	0.00	0.00	0.00
	Bin1046	0.00	5.69	63.84*	0.00	30.47	0.00	25.31	5.06	0.28	69.36*	0.00	5.89	0.00	0.00	94.11*

The ten most abundant bins of each group are presented, the dominant process is shown by *
